# Knockout of AMPKα2 Blocked the Protection of Sestrin2 Overexpression Against Cardiac Hypertrophy Induced by Pressure Overload

**DOI:** 10.3389/fphar.2021.716884

**Published:** 2021-11-17

**Authors:** Nan Zhang, Hai-Han Liao, Hong Feng, Shan-Qi Mou, Wen-Jing Li, Xiahenazi Aiyasiding, Zheng Lin, Wen Ding, Zi-Ying Zhou, Han Yan, Si Chen, Qi-Zhu Tang

**Affiliations:** ^1^ Department of Cardiology, Renmin Hospital of Wuhan University, Wuhan, China; ^2^ Cardiovascular Research Institute of Wuhan University, Wuhan, China; ^3^ Hubei Key Laboratory of Metabolic and Chronic Diseases, Wuhan, China; ^4^ Department of Geriatrics, Renmin Hospital of Wuhan University, Wuhan, China

**Keywords:** Sestrin2, cardiac hypertrophy, fibrosis, AMPKα, oxidative stress

## Abstract

**Objectives:** Sestrin2 (Sesn2) has been demonstrated to be a cysteine sulfinyl reductase and protects cells from multiple stress insults, including hypoxia, endoplasmic reticulum stress, and oxidative stress. However, the roles and mechanisms of Sesn2 in pressure overload-induced mouse cardiac hypertrophy have not been clearly clarified. This study intended to investigate whether sestrin2 (Sesn2) overexpression could prevent pressure overload-induced cardiac hypertrophy via an AMPKα2 dependent pathway through conditional knockout of AMPKα2.

**Methods and results:** Sesn2 expression was significantly increased in mice hearts at 2 and 4 weeks after aortic banding (AB) surgery, but decreased to 60–70% of the baseline at 8 weeks. Sesn2 overexpression (at 3, 6, and 9 folds) showed little cardiac genetic toxicity in transgenic mice. Cardiac dysfunctions induced by pressure overload were attenuated by cardiomyocyte-specific Sesn2 overexpression when measured by echocardiography and hemodynamic analysis. Results of HE and PSR staining showed that Sesn2 overexpression significantly alleviated cardiac hypertrophy and fibrosis in mice hearts induced by pressure overload. Meanwhile, adenovirus-mediated-Sesn2 overexpression markedly suppressed angiotensin II-induced neonatal rat cardiomyocyte hypertrophy *in vitro*. Mechanistically, Sesn2 overexpression increased AMPKα2 phosphorylation but inhibited mTORC1 phosphorylation. The cardiac protections of Sesn2 overexpression were also *via* regulating oxidative stress by enhancing Nrf2/HO-1 signaling, restoring SOD activity, and suppressing NADPH activity. Particularly, we first proved the vital role of AMPKα2 in the regulation of Sesn2 with AMPKα2 knockout (AMPKα2-/-) mice and Sesn2 transgenic mice crossed with AMPKα2-/-, since Sesn2 overexpression failed to improve cardiac function, inhibit cardiac hypertrophy and fibrosis, and attenuate oxidative stress after AMPKα2 knockout.

**Conclusion:** This study uniquely revealed that Sesn2 overexpression showed little genetic toxicity in mice hearts and inhibited mTORC1 activation and oxidative stress to protect against pressure overload-induced cardiac hypertrophy in an AMPKα2 dependent pathway. Thus, interventions through promoting Sesn2 expression might be a potential strategy for treating pathological cardiac hypertrophy and heart failure.

## 1 Introduction

Cardiac pathological hypertrophy represents a common initial stage for a variety of heart diseases caused by pathological stimuli such as hypertension-associated pressure overload, myocardial infarction-related injuries, excess neurohormonal activation, and inflammatory stimuli (Yildiz et al., 2020). Sustained pathological hypertrophy causes fetal gene re-expression, enlarged cardiomyocyte area, malignant interstitial fibrosis, and dysregulation of signaling pathways, which finally lead to heart failure. Moreover, the occurrence of heart failure means high morbidity and mortality (Murphy et al., 2020). Treatment at the initial stage of cardiac hypertrophy might block and even regress the development and progress of heart failure (Yildiz et al., 2020). However, the molecular mechanisms underlying pathological hypertrophy remain to be fully clarified.

The mechanisms involved in cardiac hypertrophy are intricated and continuous efforts have been paid to elucidate the pathways (Bauml and Underwood, 2010; Khatibzadeh et al., 2013). Among these, the AMP-activated protein kinase α (AMPKα) mammalian target of rapamycin (mTOR) pathway plays a central role in coordinating the complex signaling events (Haque and Wang, 2017; Zhang et al., 2018b). AMPK is a critical sensor and regulator of cellular energy status and exerts important functions in the intracellular adaptation to energy stress (Qi and Young, 2015). Deficiency of AMPKα or inhibiting its activity could exaggerate pathological cardiac hypertrophy, but activating AMPKα by genetic or pharmaceutical strategies could alleviate cardiac hypertrophy and improve cardiac function via suppressing excessive protein synthesis in cardiomyocytes (Ma et al., 2016; Zhang et al., 2018b). Besides, previous reports have closely correlated the AMPKα/mTOR pathway with the hypertrophic response by inhibiting oxidative stress and apoptosis (Shaw, 2009). However, some different upstream signaling and molecular mechanisms could activate or inactivate AMPKα activity depending on different pathophysiological contexts. Therefore, it is necessary to elucidate the precise mechanisms of upstream regulating molecules in different pathophysiological conditions (Qi and Young, 2015).

Sestrin2 (Sesn2), a member of the Sestrin (Sesn) family, also known as the product of hypoxia-inducible gene 95 (Hi95), is a stress-induced protein with a molecular weight of 55 kDa. Previous studies demonstrated that Sesn2 participated in various diseases by regulating apoptosis, oxidative stress, and toxicity (Kim et al., 2015c; Pasha et al., 2017; Sun et al., 2020). Sesn2 can also attenuate degenerative processes induced by aging and diabetes via depressing reactive oxygen species (ROS) accumulation and mTORC1 activation (Kim et al., 2015a). Sesn2 could exert a protective role in dopaminergic cells by maintaining autophagy activity via activating AMPK (Hou et al., 2015). However, the effects and underlying mechanisms of Sesn2 in adult mouse cardiac hypertrophy have not been clearly illustrated. Therefore, this study intends to investigate the role and mechanisms of Sesn2 in cardiac hypertrophy with Sesn2 transgenic and AMPKα2 knockout mice by establishing a pressure overload-induced cardiac hypertrophy model via aortic banding (AB) surgery.

## 2 Materials and Methods

### 2.1 Reagents

Ang II was purchased from ENZO (ALX-151-039-M025); collagenase and trypsin were purchased from Gibco (Grand Island, NY, United States); the BCA protein assay kit was bought from Pierce (Rockford, United States); and 2,7-dichlorofluorescindiacetate (DCFH-DA) was obtained from the Bioengineering Institute (Nanjing, China). The following primary antibodies were obtained from Cell Signaling Technology (CST, United States): glyceraldehyde-3-phosphate dehydrogenase (GAPDH) (#2118), p-mTORC1 (#2971), T-mTORC1 (#2983), α-actinin (#69758S), P-p70 S6 kinase (Thr389) (#9234P), T-p70 S6 kinase (#2708), P-JNK (T183/Y185) (#4668P),T-JNK (#9258), P-p44/42 MAPK (Erk1/2) (Thr202/Tyr204) (#4370P), T-ERK (#4695), P-p38 (#4511P), p38 MAPK (#9212P), T-TAK1 (#5206), P-TAK1 (#4508), T-AKT (#4691), P-AKT (#4060), acetyl-CoA carboxylase antibody (#3676), and P-acetyl-CoA carboxylase antibody (#3661S). ABCAM provided the following primary antibodies: Anti-AMPKα2 (ab3760), p-AMPKα2 (S491, ab109402), anti-SOD1 (ab16831), anti-SOD2 (ab38155), Nrf2 (ab15323), 4-hydroxynonenal (ab46545), sarcomeric α-actinin (ab68167), heme oxygenase1 (ab-13243), and NOX2/gp91phox (ab129068). The Sesn2 antibody was acquired from Proteintech (no. 10795-1-AP). Antibodies were used at 1:1,000 dilutions for Western blotting. The secondary antibodies were obtained from LI-COR Biosciences (Lincoln, United States).

### 2.2 Animals and Treatments

All animal procedures were performed following the Guidelines for the Care and Use of Laboratory Animals published by the United States National Institutes of Health (NIH Publication, revised 2011) and approved by the Animal Care and Use Committee of Renmin Hospital of Wuhan University (Protocol No. 00013274).

Sesn2 conditional manipulated transgenic mice were established according to the published protocol as shown in Supplementary Figure S1 (Deng et al., 2017; Larson and Baker, 2019; Luo et al., 2020). In brief, the CMV promoter followed by the loxP-STOP-loxP cassette was engineered to establish transgenic mice for temporal and spatial controlling of Sesn2 expression. The transgenic mice were then bred with a tamoxifen-inducible Cre mouse to obtain an inducible specific expression of Sesn2 in the cardiomyocyte after treating with tamoxifen, which was injected into the abdomen for 7 consecutive days at a dose of 20 mg/kg/d.

The AMPKα2 knockout (AMPKα2-/-) mice were described in our previous study (Deng et al., 2013). Sesn2 transgenic mice were crossed with AMPKα2-/- to test whether the protective role of Sesn2 was via activating AMPKα2. All mice used in this study (male, aged 8–10 weeks, weighing 23.5–27.5 g) were housed under specific-pathogen-free conditions with food and water available ad libitum. Transgenic mice and littermates were subjected to aortic banding (AB) or sham surgery to establish pressure overload-induced cardiac hypertrophy animal models.

### 2.3 Aortic Banding Surgery

AB surgery was performed according to our previously published surgical protocol (Liao et al., 2019). In brief, sodium pentobarbital (50 mg/kg) with intraperitoneal injection was used to anesthetize the mice. After the loss of pain stimulation reflex in mice, mice were put on a thermostatic heating pad. After the open of the left side of the chest, the thoracic aorta was exposed by blunt dissection, and the descending thoracic aorta was ligated against a 27 G needle with a 7-0 silk suture. After quickly removing the 27 G needle, the descending thoracic aorta was narrowed about 70%. The sham surgery group was performed with the same operation as described in the AB surgery process but without ligating the descending thoracic aorta. Vascular ultrasound was performed to examine the AB surgery after 1 week of AB operation. Mice with unsuccessful surgery were removed from experimental groups.

### 2.4 Cardiomyocyte Cultures

Neonatal rat cardiomyocytes (NRCMs) were isolated as previously described (Liao et al., 2019). In brief, we sacrificed the neonatal Sprague-Dawley rats (1–3 days old), cut the ventricles into pieces, and digested them with 0.125% trypsin and 0.1% collagenase type II. Then, we centrifuged the harvested cells and resuspended the sediment in 15% fetal bovine serum (FBS, GIBCO). The serum was supplemented with 100 U/ml penicillin/100 mg/ml streptomycin in case of infection and 0.1 mmol/L bromodeoxyuridine (BrdU) to inhibit the proliferation of cardiac fibroblasts. After culture at 37°C in an incubator containing 5% CO_2_ for 90 min, non-myocytes were removed and the suspended medium which consists of cardiomyocytes was seeded into 6-well culture plates.

After culture for 48 h, the cells were starved by changing the culture medium to serum-free DMEM/F12 for 6 h. Then, the cells were incubated and infected with adenoviruses of Sesn2 (Ad-Sesn2) or a similar adenovirus vector expressing the GFP protein (Ad-GFP) for 24 h. Subsequently, the infected cardiomyocytes were stimulated with Ang II (1 nM) for 24 h to induce cardiomyocyte hypertrophy. The hypertrophic phenotype was evaluated and the markers of the AMPKα/mTOR pathway were detected by RT-PCR and Western blotting. To further investigate the precise molecular mechanism of Sesn2 in cardiac hypertrophy, Ad-shAMPKα silencing AMPKα was used to further clarify the role of AMPKα in the protective role of Sesn2 in cardiac hypertrophy in NRCMs *in vitro*.

### 2.5 Echocardiography and Hemodynamics Measurements

Four weeks after AB or sham surgery, cardiac functions of mice were evaluated by echocardiography and hemodynamics as described previously (Ma et al., 2019). In brief, mice were anesthetized by inhaling 1.5% isoflurane. Mylab 30CV (Esaote S.P.A, Genoa, Italy) equipped with a 10-MHz linear array ultrasound transducer was used to examine mouse cardiac functions. The end-systolic and end-diastolic diameter of left ventricle (LVEDs and LVEDd) was measured in a parasternal short-axis view at the end of systole or diastole phase. The LV ejection fraction (EF) and fraction shortening (FS) were calculated according to LVEDs and LVEDd.

For hemodynamic measurements, a microtip catheter transducer (SPR-839, Millar Instruments, Houston, TX, United States) was inserted into the left ventricle through the right carotid artery of mice after anesthetization. The Millar Pressure-Volume System (MPVS-400, Millar Instruments) was used to record the continuous signals for the following analysis. The data were processed by PVAN data analysis software to analyze parameters, including the end-diastolic pressure (EDP), end-systolic pressure (ESP), maximal rate of pressure development (dp/dt max), and minimal rate of pressure decay (dp/dt min). After cardiac function analysis, the mice were sacrificed by decapitation. The body weight (BW), heart weight (HW), tibia length (TL), and lung weight (LW) were recorded. Then the hearts were quickly harvested and randomly divided into pathological staining and molecular analysis groups respectively. Hearts were arrested in diastole with 10% KCL, and preserved in 10% formalin for histological analysis and immunohistochemistry. Heart samples were preserved at −80°C for RT-PCR and Western blotting.

### 2.6 Histological Analysis

Mice hearts were fixed in 10% formalin for 12 h, and then were dehydrated and embedded in paraffin for cutting into 4–5 μm thick sections. The sections were stained with hematoxylin-eosin (HE) to evaluate the cross-section area (CSA) or stained with picrosirius red (PSR) to evaluate the fibrosis volume as described in our published protocol (Liao et al., 2019). After visualizing and taking photos under an optical microscope, Image Pro-Plus (version 6.0) was used to trace the outline of single cardiomyocyte to obtain cell surface area and collagen deposition area in the left ventricle for evaluating cardiac hypertrophy and fibrosis.

### 2.7 Immunohistochemistry

Immunohistochemistry was performed according to our published protocol (Wu et al., 2019). In brief, after deparaffinization and rehydration, sections were put into a 1X citrate unmasking solution for 10 min at a sub-boiling temperature (98°C). Then the sections were cooled at room temperature for 30 min. After incubation in 3% H2O2 for 10 min, sections were blocked for 1 h in a blocking solution (1X TBST/5% Normal Goat Serum) before incubation with 4-hydroxynonenal (4-HNE) overnight at 4°C. The next day, after incubation with a GTVisionTM+/HRP reagent (GK500610A, Gene tech, China), a DAB substrate kit (GK600710, Gene tech, China) was used to detect the positive area under an optical microscope for 1–10 min. After counterstaining sections with hematoxylin, we mounted sections with coverslips and took photos under an optical microscope. Image Pro-Plus (version 6.0) was used to analyze images.

### 2.8 Immunofluorescence Staining

Immunofluorescence staining was performed according to our published protocol (Zhang et al., 2018a). In brief, NRCMs were cultured on coverslips. After giving the corresponding treatment as described in the figure legends, NRCMs were washed three times with PBS, fixed with 4% paraformaldehyde, and then permeabilized with 0.2% Triton X-100. After blocking with 10% goat serum, NRCMs were incubated with a primary antibody of α-actinin (1:100) overnight at 4°C. The next day, after discarding the primary antibody, NRCMs were incubated with Alexa Fluor^®^ 488-conjugated goat anti-rabbit IgG for 1 h at 37°C. Slow Fade Gold antifade reagent with DAPI (Sigma-Aldrich) was used to stain the cell nucleus. Fluorescence images were captured by a special OLYMPUS DX51 fluorescence microscope (Tokyo, Japan) in dark conditions and were analyzed by Image-Pro Plus 6.0 software.

### 2.9 Detection of Oxidative Stress

Commercial kits (Beyotime Biotechnology, China) were used to detect the activity of SOD (Cu/Zn-SOD and Mn-SOD Assay Kit with WST-8, S0103) and NADPH oxidase (NADP+/NADPH Assay Kit with WST-8, S0179) and the malondialdehyde (MDA) (Lipid Peroxidation MDA Assay Kit, S0131S) content in fresh heart tissue (80–120 mg) according to the manufacturer’s instructions.

### 2.10 Measurements of ROS

The DCFH-DA probe (S0033S, Beyotime Biotechnology, China) was used to examine the intracellular ROS level according to the manufacturer’s instructions and our published protocol (Zhang et al., 2020). In brief, NRCMs were incubated with DCFH-DA for 2 h at 37°C, and then the cells were washed with PBS three times in the dark. Photos were taken using an Olympus IX53 fluorescence microscope. Image Pro-Plus (version 6.0) was used to analyze images.

### 2.11 RNA Isolation and Quantitative Real-Time PCR

Total RNA from the left ventricle or cultured cardiomyocytes was extracted with Trizol as previously described (Liao et al., 2019). The Transcriptor First Strand cDNA Synthesis Kit (Roche, Basel, Switzerland) was used to reverse-transcribed 2 μg of RNA into cDNA. Light Cycler 480 SYBR Green 1 Master Mix (04887352001, Roche, United States) was used to perform real-time PCR. The primers used in this study were shown in Table 1. GAPDH was used as an internal control.

**TABLE 1:** Sequences for the primers used in the qRT-PCR experiments.

Gene species	Forward (5′-3′)	Reverse (5′-3′)
Sesn2-M	AGC​AGA​GCT​GGT​TTA​GTG​AAC​CG	GAC​AAA​CCA​CAA​CTA​GAA​TGC​AGT​G
ANP-M	ATT​GAC​AGG​ATT​GGA​GCC​CAG	TCA​AGC​AGA​ATC​GAC​TGC​CTT
BNP-M	TTT​GGG​CTG​TAA​CGC​ACT​GA	CAC​TTC​AAA​GGT​GGT​CCC​AGA
αMHC-M	AGG​TGG​ACC​TGA​TCA​TGG​AG	ATA​CCG​GAG​ATC​ATG​CAA​GC
βMHC-M	CCG​AGT​CCC​AGG​TCA​ACA​A	CTT​CAC​GGG​CAC​CCT​TGG​A
Collagen I-M	AGC​ACG​TCT​GGT​TTG​GAG​AG	GAC​ATT​AGG​CGC​AGG​AAG​GT
Collagen III-M	TGA​CTG​TCC​CAC​GTA​AGC​AC	GAG​GGC​CAT​AGC​TGA​ACT​GA
CTGF-M	AGA​CCT​GTG​CCT​GCC​ATT​AC	ACG​CCA​TGT​CTC​CGT​ACA​TC
Fibronectin-M	GAC​CCT​TAC​ACG​GTT​TCC​CA	AAG​CAC​TGG​CAT​GTG​AGC​TT
αSMA-M	CCA​GCC​ATC​TTT​CAT​TGG​GAT	ACA​GGA​CGT​TGT​TAG​CAT​AGA​G
GAPDH-M	ACT​CCA​CTC​ACG​GCA​AAT​TC	TCT​CCA​TGG​TGG​TGA​AGA​CA
Sesn2-H	TGC​TTA​ATG​GTG​TGA​GGC​GT	GGC​AAT​GTG​ACC​AGC​AAA​GG
ANP-R	CGG​TAC​CGA​AGA​TAA​CAG​CCA	TCA​CCA​CCT​CTC​AGT​GGC​AA
βMHC-R	AGT​GAA​GAG​CCT​CCA​GAG​TTT​G	GTT​GAT​GAG​GCT​GGT​GTT​CTG​G
Collagen I-R	GAG​AGA​GCA​TGA​CCG​ATG​GAT​T	TGG​ACA​TTA​GGC​GCA​GGA​A
Collagen III-R	AAG​GGC​AGG​GAA​CAA​CTG​AT	GTG​AAG​CAG​GGT​GAG​AAG​AAA​C
CTGF-R	AGA​CAC​ATT​TGG​CCC​TGA​CC	TCT​TAG​AAC​AGG​CGC​TCC​AC
Fibronectin-R	GGA​TCC​CCT​CCC​AGA​GAA​GT	GGG​TGT​GGA​AGG​GTA​ACC​AG
αSMA-R	CAT​CAC​CAA​CTG​GGA​CGA​CA	TCC​GTT​AGC​AAG​GTC​GGA​TG
GAPDH-R	GAC​ATG​CCG​CCT​GGA​GAA​AC	AGC​CCA​GGA​TGC​CCT​TTA​GT

M, mouse; R: rat; H, human.

### 2.12 Western Blotting Analysis

Total proteins were extracted according to published protocols (Liao et al., 2019). In brief, heart tissue or NRCMs was lysed in RIPA lysis buffer. The protein concentration was measured by using the BCA Protein Assay Kit (23227, Thermo Scientific, China). Protein lysates were electrophoresed in different concentrations of SDS-PAGE (8, 10, and 12%), and then transferred onto PVDF membranes. After blocking the membranes with 5% BSA for 1 h, the blots were incubated with primary antibodies overnight at 4°C. The next day, blots were incubated with secondary antibodies at room temperature for 1 h. All blots were visualized using ChemiDoc TM XRS + (Bio-Rad). The blots were quantified and analyzed by using Image Lab software.

### 2.13 Statistical Analysis

All of the data in our study were expressed as Mean ± SD (standard deviation). Cell experiments were repeated three times independently. The experiments and analysis were blinded whenever it need. SPSS 22.0 software was used to data analysis. One-way analysis of variance (ANOVA) and Tukey post hoc tests were used for multi-group comparisons. *p* < 0.05 was considered statistically significant.

## 3 Results

### 3.1 The Expression of Sesn2 in Hypertrophic Hearts and Cardiomyocytes

**FIGURE 1:**
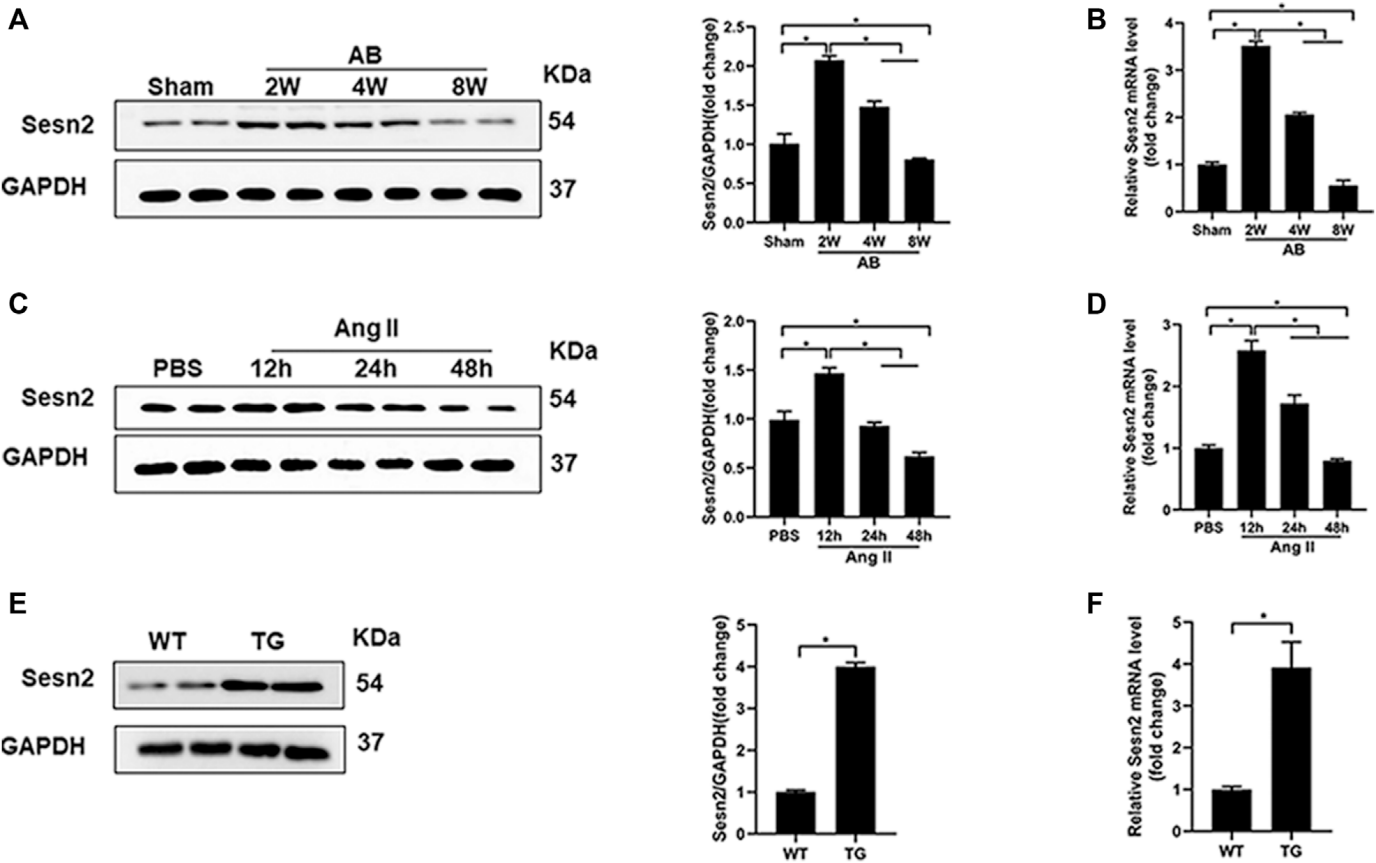
The expression of Sesn2 in hypertrophic hearts and Ang II-induced cardiomyocytes. **(A)** Representative immunoblots and quantitative results of Sesn2 protein expression in mice hearts at 2, 4, and 8 weeks after AB surgery (*n* = 6). **(B)** qPCR analysis of the mRNA levels of Sesn2 at 2, 4, and 8 weeks after AB surgery (*n* = 6). **(C)** Representative Western blots and statistical analysis of Sesn2 expression in NRCMs at 12, 24, and 48 h after Ang II treatment (*n* = 6). **(D)** Sesn2 mRNA expression levels in NRCMs treated with or without Ang II stimulation (*n* = 6). **(E)** Representative Western blots and statistical analysis of Sesn2 expression in the hearts of WT and TG mice, respectively (*n* = 6). **(F)** Sesn2 mRNA expression in WT and TG mice hearts (*n* = 6). The data were expressed as the mean ± SD.*p < 0.05 *vs.* indicated group. One-way analysis of variance (ANOVA) followed by Tukey post hoc tests were used for significance analysis.

As shown in Figures 1A,B, results of Western blotting and RT-PCR exhibited that Sesn2 expression was significantly upregulated in mice hearts 2 and 4 weeks after AB surgery compared to the sham-operated group, while the expression level reduced to 60–70% of the baseline after 8 weeks of AB surgery. In isolated NRCMs, angiotensin II (Ang II 1 μM) treatment caused a significant upregulation of Sesn2 at 12 h compared to the PBS treatment group both at protein and mRNA level. However, Ang II treatment for 48 h significantly decreased Sesn2 expression compared to the PBS treatment group (Figures 1C,D). These results implied that Sesn2 might take part in regulating pathological cardiac hypertrophy. To evaluate the role of Sesn2 in cardiac hypertrophy, transgenic mice (TG) were constructed for human Sesn2 specific overexpression in the cardiomyocytes (Supplementary Figure S1). The human Sesn2 was successfully overexpressed in the hearts of TG (Figures 1E,F). The TG mice and their wild-type (WT) littermates were used for the following experiments.

### 3.2 Sesn2 Overexpression in Cardiomyocytes Alleviated Pressure Overload Induced Cardiac Dysfunction

**FIGURE 2:**
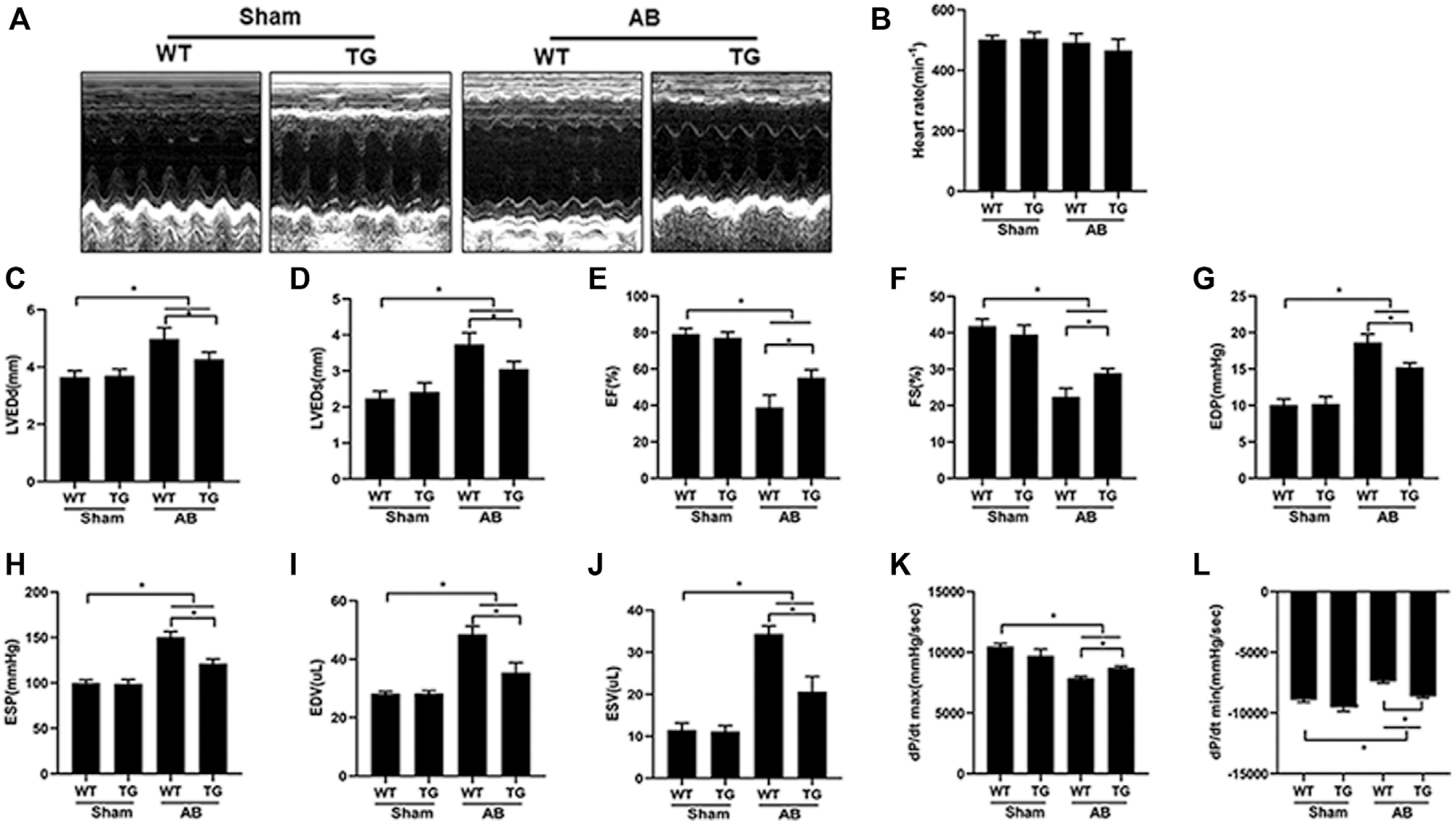
Cardiomyocyte-specific overexpression of Sesn2 attenuated pressure overload-induced cardiac dysfunction. **(A)** Representative echocardiographic images in each group (*n* = 6). Echocardiography and pressure-volume loop were performed to evaluate mouse cardiac function at 8 weeks after AB surgery (*n* = 6). **(B)** heart rate (HR), **(C)** left ventricular end diastolic diameter (LVEDd), **(D)** left ventricular end systolic diameter (LVEDs), **(E)** left ventricular ejection fraction (EF), **(F)** LV fraction shortening (FS), **(G)** end diastolic pressure (EDP), **(H)** end systolic pressure (ESP), **(I)** end diastolic volume (EDV), **(J)** end systolic volume (ESV), **(K)** left ventricular maximal rate of pressure rise (dp/dt max), **(L)** left ventricular maximal rate of pressure decay (dp/dt min). Data were presented as the mean ± SD. *p < 0.05 vs indicated group. One-way analysis of variance (ANOVA) followed by Tukey post hoc tests were used for significance analysis.

Echocardiography was performed to examine mouse cardiac function among different groups (Figure 2A). There was no difference in heart rate among all groups (Figure 2B). Four weeks of pressure overload caused a marked increase in parameters, including left ventricular end-diastolic diameter (LVEDd) (Figure 2C), left ventricular end-systolic diameter (LVEDs) (Figure 2D), end diastolic pressure (EDP) (Figure 2G), end systolic pressure (ESP) (Figure 2H), end diastolic volume (EDV) (Figure 2I), and end systolic volume (ESV) (Figure 2J), but significantly decreased left ventricular ejection fraction (EF) (Figure 2E) and left ventricular fraction shortening (FS) (Figure 2F) in the WT + AB group, compared to WT + sham group. However, Sesn2 overexpression markedly alleviated cardiac dysfunction induced by pressure overload, as evidenced by preserved LVEF and FS, decreased LVEDd, LVEDs, EDP, ESP, EDV, and ESV in the TG + AB group compared to the WT + AB group. Pressure volume loop analysis was used to further assess the mouse cardiac function. Pressure overload caused a significant decrease in maximal left ventricular pressure rising rate (dp/dt max) and the rate of left ventricle diastolic pressure change (dp/dt min) in the WT + AB group compared to the WT + sham group (Figures 2K,L). Similarly, Sesn2 overexpression significantly restored the dp/dt max and dp/dt min in the TG + AB group compared to the WT + AB group (Figures 2K,L), which confirmed again the protective role of Sesn 2 in cardiac dysfunction caused by pressure overload.

### 3.3 Sesn2 Overexpression Improved Pressure Overload-Induced Cardiac Hypertrophy and Fibrosis in Mice

**FIGURE 3:**
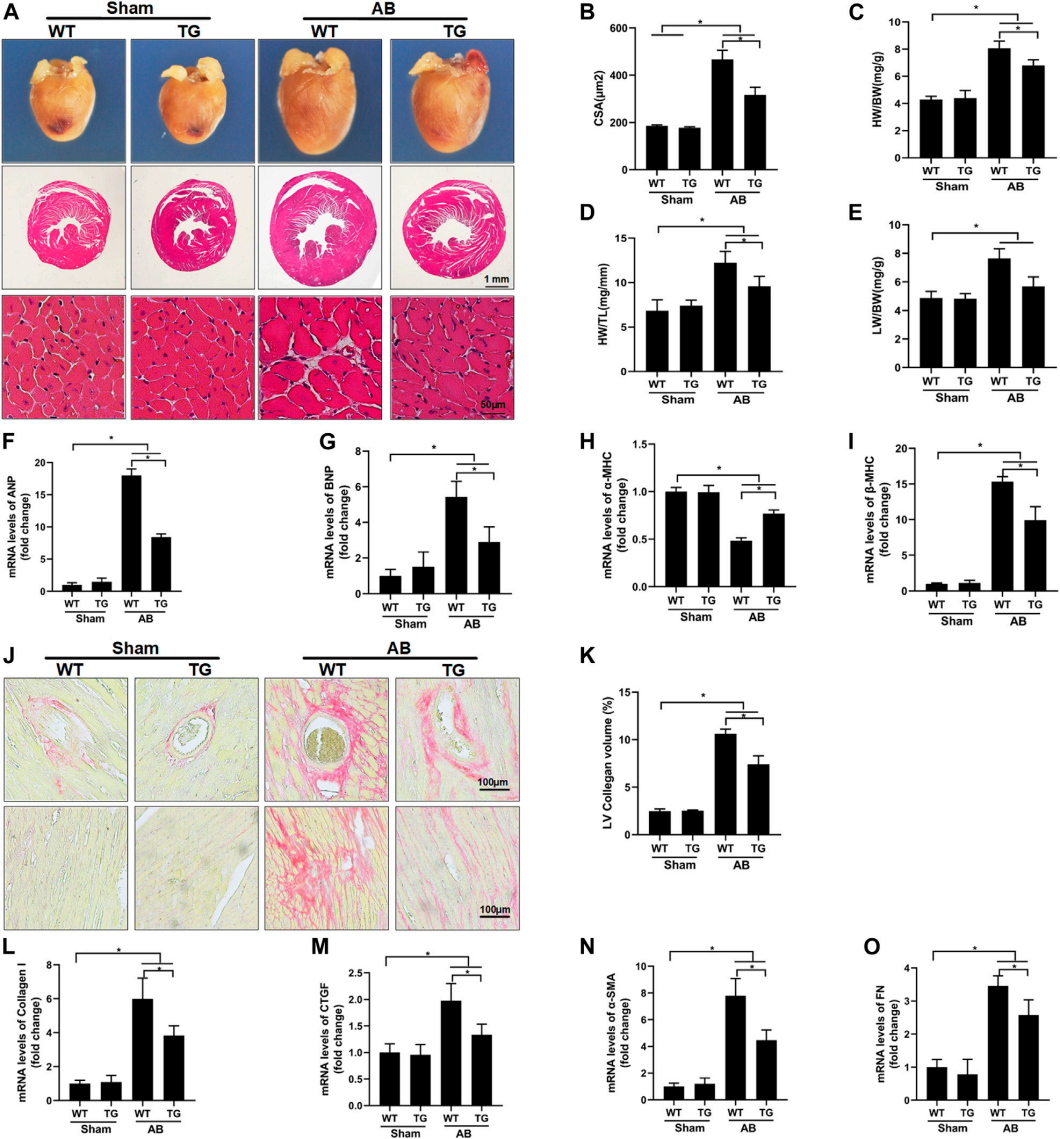
Sesn2 overexpression alleviated pressure overload-induced hypertrophy and fibrosis in mice. **(A)** Representative images of gross hearts and HE staining in the indicated groups (*n* = 6). **(B)** Quantification of the cross-sectional area (CSA) of cardiomyocytes in the indicated mice (*n* ≥ 100 left ventricular cells). Statistical results of the **(C)** HW/BW, **(D)** HW/TL **(E)** and LW/BW of mice at 8 weeks after AB surgery(*n* = 9). Cardiac mRNA levels of. **(F)** ANP, **(G)** BNP, **(H)** α-MHC, and **(I)** β-MHC. **(J)** Picrosirius red (PSR) staining of histological sections of left ventricles (*n* = 6), **(K)** quantitative results of the left ventricular collagen volume (*n* ≥ 25 fields). L-O The mRNA levels of **(L)** Collagen I, **(M)** CTGF, **(N)** α-SMA, and **(O)** FN. ANP: atrial natriuretic peptide; BNP, brain natriuretic peptide; α-MHC, α-myosin heavy chain; β-MHC, β-myosin heavy chain; HW, heart weight; BW, body weight; TL, tibia length; HE, hematoxylin and eosin; CTGF, connective tissue growth factor; FN, fibronectin; αSMA, alpha-smooth muscle actin (αSMA). The data were expressed as the mean ± SD. *p < 0.05 *vs.* indicated group. One-way analysis of variance (ANOVA) followed by Tukey post hoc tests were used for significance analysis.

Pressure overload significantly induced mouse heart hypertrophy, as shown by the increased cardiomyocyte surface area (CSA) in the WT + AB group compared to the WT + sham group. Sesn2 overexpression inhibited cardiac hypertrophy, since it decreased CSA in the TG + AB group compared to the WT + AB group (Figures 3A,B). Congruously, the heart weight/body weight (HW/BW) (Figure 3C), heart weight/tibia length (HW/TL) (Figure 3D), and lung weight/body weight (LW/BW) (Figure 3E) were significantly increased in the WT + AB group compared to the WT + sham group. Sesn2 overexpression significantly decreased HW/BW, HW/TL, and HW/BW in the TG + AB group compared to the WT + AB group (Figures 3C–E). The hypertrophic markers, including atrial natriuretic peptide (ANP) (Figure 3F), B-type natriuretic peptide (BNP) (Figure 3G), and β-MHC (Figure 3I), were significantly upregulated in the WT + AB group compared to the WT + sham group. However, Sesn2 overexpression significantly depressed the mRNA expression of these hypertrophic markers in the TG + AB group compared to the WT + AB group (Figures 3F,G,I). Besides, pressure overload caused a significant decrease of α-MHC in the WT + AB group compared to the WT + sham group, but Sesn2 overexpression obviously reversed the α-MHC expression in the TG + AB group compared to the WT + AB group (Figure 3H). Taken together, these data exhibited that Sesn2 overexpression protected against pressure overload-induced cardiac hypertrophy.

Cardiac fibrosis is an integrated process of pathological cardiac hypertrophy. PSR staining revealed that pressure overload induced significant fibrosis around the perivascular and in the interstitium of the mouse heart in the WT + AB group compared to the WT + sham group, but Sesn2 overexpression markedly mitigated cardiac fibrosis in the TG + AB group compared to the WT + AB group (Figures 3J,K). Moreover, the fibrosis associated markers, including collagen I (Figure 3L), connective tissue growth factor (CTGF) (Figure 3M), alpha-smooth muscle actin (α-SMA) (Figure 3N), and fibronectin (FN) (Figure 3O), were significantly upregulated in the WT + AB group compared to the WT + sham group. However, Sesn2 overexpression depressed the expression of these fibrosis-associated markers in the TG + AB group compared to the WT + AB group (Figures 3L–O). Therefore, cardiac fibrosis caused by pressure overload could also be ameliorated by Sesn2.

### 3.4 Sesn2 Overexpression Activated the AMPKα2 Signaling Pathway and Suppressed Oxidative Stress

**FIGURE 4:**
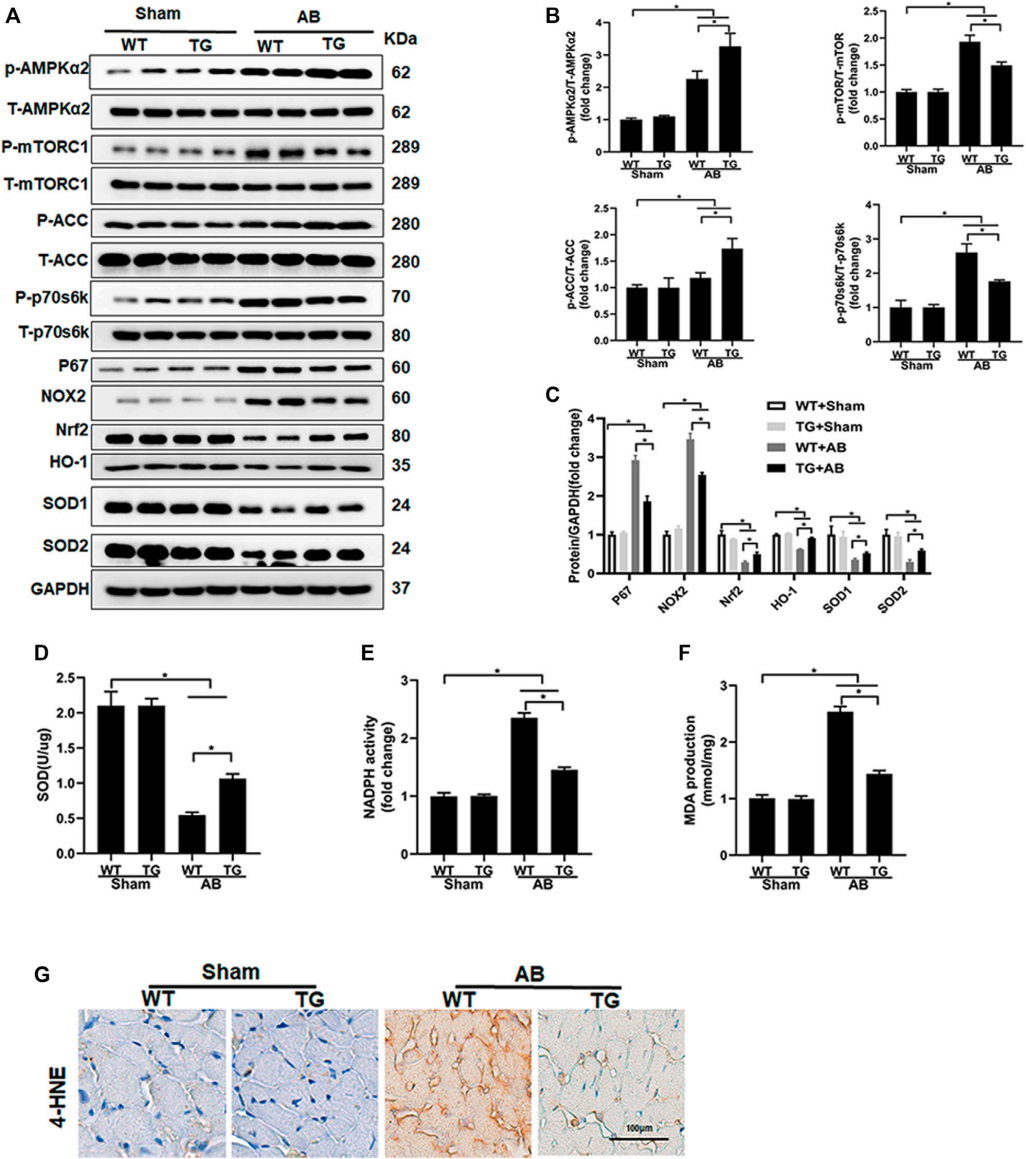
Sesn2 overexpression activated the AMPKα2 pathway and suppressed oxidative stress. **(A)** Representative Western blots of the phosphorylated and total protein expression of AMPKα2, mTORC1, ACC, P70s6k, P67, NOX2, Nrf2, HO-1, SOD1, and SOD2 at 8 weeks after AB surgery (*n* = 6). **(B,C)** Quantification of the proteins. **(D)** Examination of SOD activity (*n* = 6), **(E)** Examination of NADPH activity (*n* = 6), **(F)** Examination of MDA accumulation (*n* = 6). **(G)**. Immunohistochemical staining for 4-hydroxynonenal (4-HNE). *p < 0.05 *vs.* indicated group. One-way analysis of variance (ANOVA) followed by Tukey post hoc tests were used for significance analysis.

In previous studies, Sesn2 has been suggested to regulate the MAPK, AKT, AMPKα, and oxidative stress-associated signaling pathways, all of which have been implicated in the regulation of pathological cardiac hypertrophy. Our data presented that Sesn2 overexpression seemed not to regulate the MAPK and AKT signaling pathway (Supplementary Figures S2A,B). However, AMPKα2 and ACC phosphorylation was significantly enhanced by Sesn2 overexpression in the TG + AB group compared to the WT + AB group. Meanwhile, mTORC1, and p70s6k phosphorylation was significantly inhibited in the TG + AB group compared to the WT + AB group (Figures 4A,B).

We also detected the oxidative stress-associated signaling pathway and oxidative stress status among different groups. The pro-oxidative stress markers, including p67 and NOX2, and the anti-oxidative stress proteins, including Nrf2, HO-1, SOD1, and SOD2 were significantly upregulated and downregulated in the WT + AB group compared to the WT + sham group, respectively (Figure 4A). Sesn2 overexpression markedly suppressed the expression of p67 and NOX2, and restored the expression of Nrf2, HO-1, SOD1, and SOD2 in the TG + AB group compared to the WT + AB group (Figures 4A,C). SOD catalyzes the conversion of superoxide radicals into hydrogen peroxide and oxygen, which could prevent cells from ROS-associated damage. The SOD activity was significantly decreased but the NADPH activity was markedly increased in the WT + AB group compared to the WT + sham group, which could be restored by Sesn2 overexpression in the TG + AB group compared to the WT + AB group (Figures 4D,E). Finally, the products of oxidative stress, including MDA and 4-hydroxynonenal (4-HNE), were significantly accumulated in the WT + AB group compared to the WT + sham group, which could be markedly inhibited by Sesn2 overexpression in the TG + AB group compared to the WT + AB group (Figures 4F,G).

### 3.5 Sesn2 Inhibited Cardiomyocyte Hypertrophy *in vitro*

**FIGURE 5:**
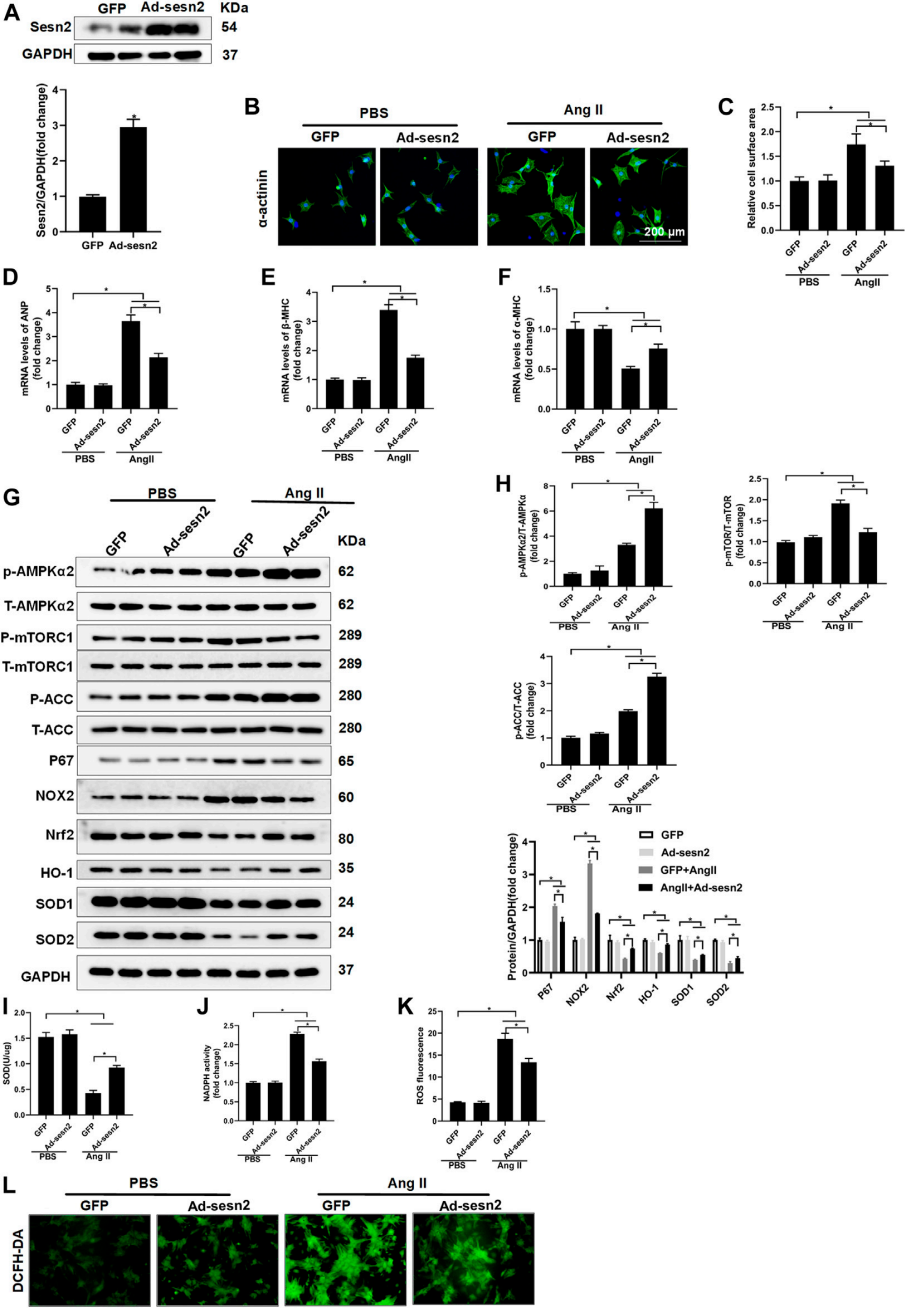
Sesn2 overexpression alleviates Ang II- induced cardiomyocyte hypertrophy *in* vitro. NRCMs were transfected with adenovirus (Ad)-Sesn2 or Ad-GFP for 24 h, and then treated with Ang II for another 48 h. **(A)** Examination of Sesn2 overexpression in cardiomyocytes (*n* = 6). **(B,C)** Representative images of α-actinin staining and statistical results of cell surface area (*n* > 50 cells per group). mRNA expression of **(D)** ANP, **(E)** α-MHC and **(F)** β-MHC. **(G)** Representative Western blots of the phosphorylated and total proteins of AMPKα2, mTORC1, ACC, P67, NOX2, Nrf2, HO-1, SOD1, and SOD2 in NRCMs transfected with Ad-Sesn2 or Ad-GFP for 24 h and treated with Ang II for 48 h (*n* = 6). **(H)** Quantitative results of the Western blotting analysis. Examination of **(I)** SOD and **(J)** NADPH activities transfected with Ad-Sesn2 or Ad-GFP for 24 h and treated with Ang II for 48 h (*n* = 6). **(K,L)** DCFH-DA staining and ROS calculation in NRCMs transfected with Ad-Sesn2 or Ad-GFP and treated with Ang II for 48 h (*n* = 6). NRCM: neonatal rat cardiomyocytes. DCFH-DA: DCFH-DA; 2′,7′-dichlorodihydrofluorescein diacetate. The data were expressed as the mean ± SD from 3 independent experiments. *p < 0.05 *vs.* indicated group. One-way analysis of variance (ANOVA) followed by Tukey post hoc tests were used for significance analysis.

NRCMs were isolated and transfected with adenovirus for Sesn2 (Ad-Sesn2) overexpression in order to investigate the role of Sesn2 in Ang II-induced cardiomyocyte hypertrophy. As shown in Figure 5A, Sesn2 was successfully overexpressed in the NRCMs. Ang II treatment for 48 h successfully induced cardiomyocyte hypertrophy *in vitro*, as evidenced by significantly enlarged cardiomyocyte surface area (Figures 5B,C), markedly increased expression of ANP and β-MHC, and dramatically decreased α-MHC in the GFP + Ang II group compared to the GFP + PBS group. In accordance with results from experiments *in vivo*, the Sesn2 overexpression significantly inhibited cardiomyocyte hypertrophy, suppressed mRNA expression of ANP and β-MHC, and restored mRNA expression of α-MHC in the Ad-Sesn2 + Ang II group compared to the GFP + Ang II group (Figures 5D–F).

Sesn2 overexpression significantly promoted the phosphorylation of AMPKα2 and ACC, and decreased mTORC1 phosphorylation in NRCMs treated with Ang II (Figures 5G,H). The expression of Nrf2, HO-1, SOD1, and SOD2 was obviously restored but the expression of p67 and NOX2 was reduced in the AngII + Ad-Sesn2 group compared to the Ang II + GFP group (Figures 5G,H). Besides, the activity of SOD was significantly reduced while the activity of NADPH was significantly increased in the Ang II + GFP group compared to the PBS + GFP group. However, Sesn2 overexpression successfully inhibited Ang II-induced increase in NADPH activity but enhanced the SOD activity (Figures 5I,J). In addition, we detected the ROS production in each group and found that Sesn2 overexpression dramatically inhibited ROS over-production induced by Ang II (Figures 5K,L).

### 3.6 Sesn2 Overexpression Failed to Protect Against Cardiac Dysfunction and Cardiac Hypertrophy in AMPKα2 Knockout Mice

**FIGURE 6:**
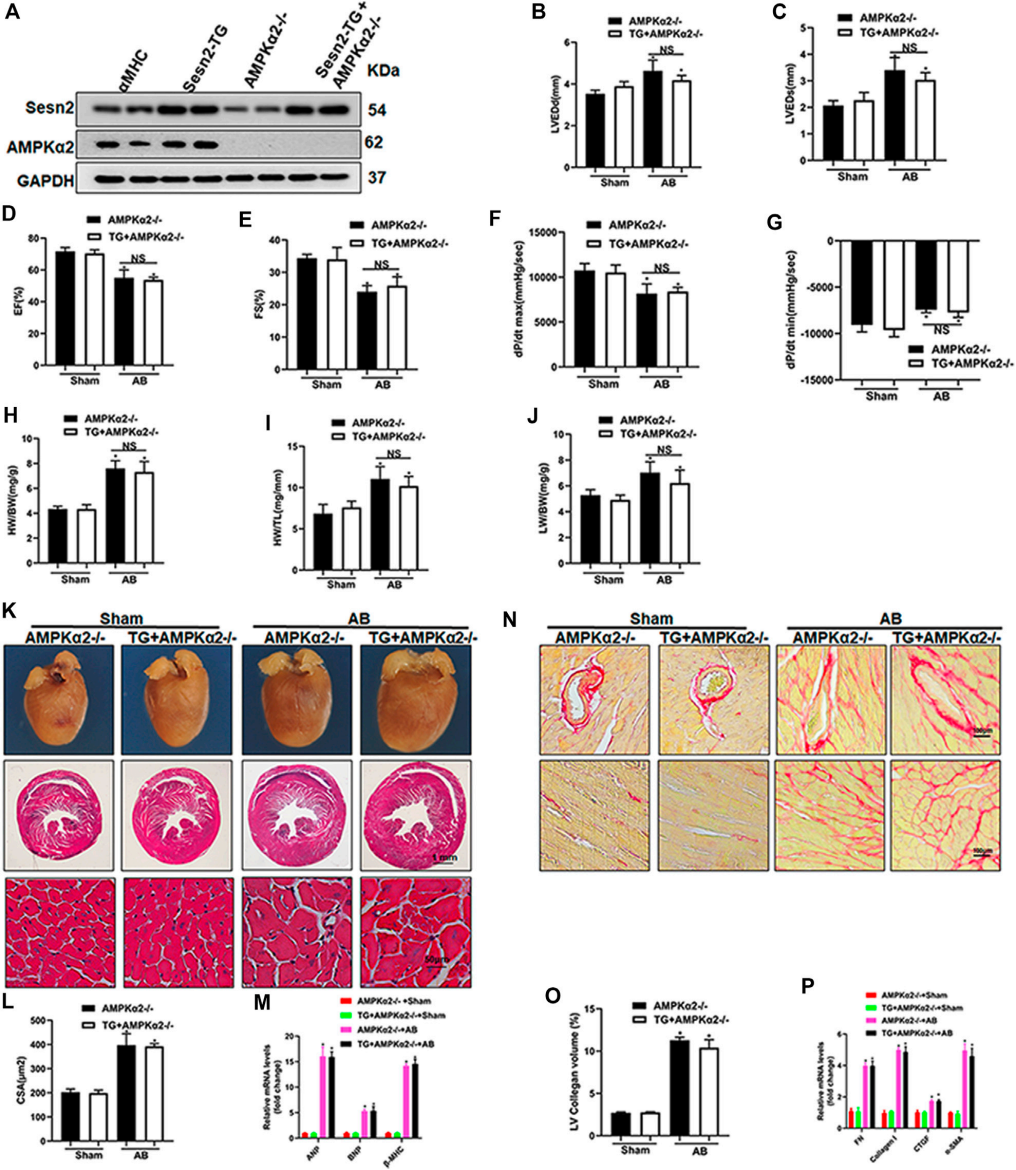
Sesn2 overexpression could not protect against cardiac remodeling in AMPKα2 knockout mice. **(A)** Representative Western blots of Sesn2 and AMPKα2 (*n* = 6). Echocardiography and pressure-volume loop parameters detected mouse cardiac function at 8 weeks after sham or AB surgery (*n* = 6): **(B)** LVEDd, **(C)** LVEDs, **(D)** EF, **(E)** FS, **(F)** dp/dtmax and **(G)** dp/dtmin. The ratios of **(H)** HW/BW, **(I)** HW/TL and **(J)** LW/BW at 8 weeks after sham or AB surgery (*n* = 9). **(K)** Representative images of gross hearts and HE-stained heart sections (*n* = 6). **(L)** Quantitative measurements of cell surface area (CSA) of cardiomyocytes (*n* = 60) (*n* ≥ 100 left ventricular cells). **(M)** RT-PCR detected ANP, BNP and βMHC mRNA expression (*n* = 6). **(N)** Representative images of PSR staining (*n* = 6). **(O)** Quantitative results of the left ventricular collagen volume (*n* = 6). **(P)** RT-PCR detected FN, Collagen I, CTGF, and α-SMA mRNA expression. The data were expressed as the mean ± SD. *p < 0.05 *vs.* indicated group. One-way analysis of variance (ANOVA) followed by Tukey post hoc tests were used for significance analysis.

To further determine whether the cardiac protective effect of Sesn2 overexpression was dependent on AMPKα2 signaling pathway, we crossed the cardiac-specific overexpression of Sesn2 mice (Sesn2-TG) with AMPKα2 knockout mice (AMPKα2-/-) to construct Sesn2-TG + AMPKα2-/- mice. As shown in Figure 6A, Western blotting analysis was performed to examine the expression of Sesn2 and AMPKα2 in Sesn2-TG + AMPKα2-/- mice. Pressure overload caused mouse cardiac dysfunction, as evidenced by significantly increased LVEDd and LVEDs and decreased EF and FS in the AMPKα2-/- + AB group compared to the AMPKα2-/- + sham group (Figures 6B–E). However, no significant difference on these parameters could be detected between the Sesn2-TG + AMPKα2-/- + AB group and the AMPKα2-/- + AB group (Figures 6B–E). Pressure volume-loop analysis also showed that pressure overload could significantly disturb the dp/dt max and dp/dt min (Figures 6F,G) in the AMPKα2-/- + AB group compared to the AMPKα2-/- + sham group, but no significant difference could be detected on dp/dt max and dp/dt min between the Sesn2-TG + AMPKα2-/- + AB group and the AMPKα2-/- + AB group. These data show that Sesn2 overexpression failed to prevent mouse cardiac dysfunction in AMPKα2-/- mice.

Parameters including HW/BW, HW/TL, and LW/BW increased in the AMPKα2-/- + AB group compared to the AMPKα2-/- + sham group, while there was no significant difference on these parameters between the Sesn2-TG + AMPKα2-/- + AB group and the AMPKα2-/- + AB group (Figures 6H–J). HE staining exhibited pressure overload induced increased cardiomyocyte surface area, which could not be inhibited by Sesn2 overexpression in AMPKα2-/- mice (Figures 6K,L). Meanwhile, the mRNA expression of ANP, BNP, and β-MHC in AMPKα2-/- + AB group was increased when compared to the AMPKα2-/- + sham group, but Sesn2 overexpression showed little effect on ANP, BNP, and β-MHC expression in the Sesn2-TG + AMPKα2-/- + AB group compared to the AMPKα2-/- + AB group (Figure 6M).

In addition, PSR staining showed that pressure overload induced significant fibrosis in interstitium and peri-vascular heart tissue, which could not be inhibited by Sesn2 overexpression in AMPKα2-/-mouse (Figures 6N,O). Similarly, the mRNA expression of fibrosis markers, including FN, collagen I, CTGF, and α-SMA, were significantly increased in the AMPKα2-/- + AB group compared to the AMPKα2-/- + sham group. However, Sesn2 overexpression failed to block the increase of FN, Collagen I, CTGF, and α-SMA expression in AMPKα2 subjected to AB surgery (Figure 6P). To sum up, these data showed that AMPKα2 deficiency completely abrogated the protective effects of Sesn2 overexpression. Furthermore, in AMPKα2-/- mice, Sesn2 overexpression could not inhibit Nox2 or p67 expression and restore Nrf2, HO-1, SOD1, and SOD2 expression (Supplement Figures S3A,B).

### 3.7 Ang II-Induced Hypertrophy and Oxidative Stress Could Not Be Prevented by Sesn2 Overexpression After AMPKα2 Silence in NRCMs

NRCMs were transfected with Ad-Sesn2 or Ad-shAMPKα2 to over-express Sesn2 and silence AMPKα2 respectively, as shown in Supplement Figures S4A,B. The phosphorylation of AMPKα2 was downregulated by about 90%, and Sesn2 was enhanced about 3.5 fold compared with the control group. Ang II treatment significantly increased the cell surface of NRCMs and promoted mRNA expression of ANP and β-MHC (Supplement Figures S4C–S4F). There was no significant difference on CSA and mRNA expression of ANP and β-MHC between the Ang II + Ad-Sesn2 + Ad-shAMPKα2 group and the Ang II + Ad-shAMPKα2 group (Supplement Figures S4C–S4F). Moreover, Ang II treatment caused a significant accumulation of ROS, which could not be inhibited by Sesn2 overexpression in NRCMs after AMPKα2 silence (Supplement Figure S4G). Finally, we also presented that Ang II treatment decreased SOD activity and enhanced NADPH activity in the Ang II + Ad-shAMPKα2 group compared to the Ad-shAMPKα2 group. However, Sesn2 overexpression showed no effects on regulating SOD and NADPH activity in NRCMs after AMPKα2 silence (Supplement Figures S4H,I).

## 4 Discussion

We firstly demonstrated that Sesn2 expression was upregulated at 2 weeks and decreased to 60% of the baseline at 8 weeks after AB surgery. A previous study indicated that Sesn2 was significantly downregulated at the end of 10 weeks after transverse aortic constriction surgery (Du et al., 2019). These studies implied that Sesn2 might take part in regulating pressure overload-induced pathological cardiac hypertrophy. We established a transgenic (TG) mouse for specifically overexpressing Sesn2 in cardiomyocytes by crossing with a transgenic mouse with α-MHC mediated Cre expression. The TG mouse subjected to aortic banding surgery revealed that Sesn2 overexpression prevented mice hearts from pressure overload-induced cardiac dysfunction, hypertrophy, and fibrosis. The underlying mechanisms might be at least partly through activating AMPKα2, inhibiting mTORC1/p70s6k signaling pathway, and suppressing excessive oxidative stress *via* restoring Nrf2/HO-1 and depressing NOX and NAPDH activity. These mechanisms could also be confirmed in Ang II treated NRCMs *in vitro*. Moreover, this study demonstrated that Sesn2 overexpression failed to protect against pressure overload-induced pathological hypertrophy in AMPKα2-/- mouse and NRCMs with AMPKα2 silence. Thus, this study firstly demonstrated that transgenic Sesn2 overexpression negatively regulated cardiac hypertrophy and combating oxidative stress, which was dependent on AMPKα2 regulation.

AMPK is a heterotrimeric protein containing a catalytic subunit (α) and two regulatory subunits (β and γ) (Zaha and Young, 2012). The α subunit has two isoforms (α1 and α2) encoded by different genes and the α2 isoform mainly exists in cardiomyocytes (Zaha and Young, 2012). AMPK functions as the hub protein of regulating fatty acid oxidation (FAO) in the cardiomyocytes by supplying ATP (Zuurbier et al., 2020). AMPK activation could phosphorylate and depress the activity of both isoforms of acetyl-CoA carboxylase (AAC1/ACC2), which suppresses the conversion of acetyl-CoA to malonyl-CoA (Zuurbier et al., 2020). Malonyl-CoA is a rate-limiting enzyme for FAO (Zuurbier et al., 2020), and the accumulation of malonyl-CoA could inhibit fatty acid uptake of mitochondria by inhibiting CPT1 activity and result in FAO inhibition (Zuurbier et al., 2020). Activating AMPKα2 has been suggested to promote FAO for alleviating pathological hypertrophy (Zuurbier et al., 2020). Besides, multiple hypertrophic stimuli, including pressure overload, β-adrenergic stimulation, angiotensin II and IGF-1, could cause mTORC1 hyperphosphorylation, and lead to exaggerated pathological cardiac hypertrophy (Sciarretta et al., 2018). Inhibiting mTORC1 activity by activating AMPKα2 could effectively protect against cardiac hypertrophy induced by various pro-hypertrophic stimuli (Sciarretta et al., 2018). However, AMPKα2 deletion caused malignant activation of the mTORC1 signaling pathway, resulting in exacerbated cardiac hypertrophy and dysfunction (Zhang et al., 2008). Direct inhibition and indirect inhibition by AMPKα2 activation of mTORC1 significantly mitigated cardiac hypertrophy (Zhang et al., 2008). These studies clearly indicated that mTORC1 inhibition through activating AMPKα2 might be a potential strategy for protecting against pressure overload or neurohumoral factors induced pathological cardiac hypertrophy. Discovering new targets for regulating AMPK/mTOR signaling pathway might efficiently inhibit the development and progress of cardiac hypertrophy and heart failure.

Previous studies found that genotoxic stress could induce p53-dependent Sesn2 upregulation. Upregulated Sesn2 interacted with TSC1:TSC2 and AMPKα for activating AMPKα phosphorylation, which led to the inhibition of mTROC1 signaling (Budanov and Karin, 2008). Sesn2 deficiency exaggerated hyper-nutrition and obesity-associated insulin resistance and hepato-steatosis through chronic activation of mTORC1-p70/S6K signaling, which could be effectively reversed by metformin treatment via activating AMPKα (Lee et al., 2012). Sesn2 overexpression mitigated rotenone-induced α-synuclein accumulation and caspase 3 activation via enhancing AMPK-dependent autophagy in dopaminergic cells (Hou et al., 2015). Lee et al. reported that loss of drosophila sestrin (dSesn) resulted in age-associated cardiac malfunction, which could be prevented by pharmacological activation of AMPK or inhibition of mTOR (Lee et al., 2010). These studies presented that Sesn2 inhibited mTORC1 activity via an AMPK-dependent pathway.

Some other studies also presented that Sesn2 inhibited mTORC1 phosphorylation via an AMPK-independent pathway. Park HW et al. demonstrated that Sesn2 expression could be induced by an endoplasmic reticulum (ER) stress-activated transcription factor in the liver (Park et al., 2014). Once induced, Sesn2 could depress protein synthesis by inhibiting mTORC1 through an AMPK-independent manner (Park et al., 2014). Parmigiani A et al. demonstrated that Sesn2 could inhibit mTORC1 activation via the interaction with GTPase-activating protein activity toward Rags 2 (GATOR2) instead of an AMPK-dependent mechanism (Parmigiani et al., 2014; Kim et al., 2015b). Lately, Nanhu Quan et al. reported that Sesn2 deficiency exaggerated pressure overload or age-induced mouse cardiac hypertrophy and dysfunction (Quan et al., 2020), and adeno-associated virus 9-mediated Sesn2 overexpression in the mouse heart could attenuate these changes *via* interacting with GATOR2 and thus inhibiting mTORC1 activity (Quan et al., 2020). Therefore, these studies showed that Sesn2 might regulate mTORC1 activation through an AMPK-independent but GATOR2-dependent pathway. However, relying on AMPKα2-/- mice, our study firstly demonstrated that the inhibition of Sens2 on mTORC1/p70s6k activation was via an AMPK-dependent manner in pressure overload-induced pathological cardiac hypertrophy, because Sesn2 overexpression could not depress mTORC1/p70s6K signaling pathway in AMPKα2-/- mouse heart.

Besides the AMPK/mTORC1 pathway, Sesn2 has been suggested to regulate signals involved in oxidative stress in various diseases. Sesn2 expression was significantly elevated in peripheral nerves after injuries. Sesn2 knockout mice presented extraordinarily augmented late-phase neuropathic pain behavior because of excessive ROS accumulation (Kallenborn-Gerhardt et al., 2013). The analysis of the crystal structure of human Sesn2 demonstrated that a structure of helix-turn-helix oxidoreductase motif locating at the N-terminal domain inhibited ROS production (Kim et al., 2015a). Forced expression of Sesn2 could induce keap1 degradation via a p62-dependent pathway, resulting in upregulation of Nrf2 activity, thus protect the mouse liver from acute stimulation of lipogenesis-associated oxidative damage (Bae et al., 2013). Sesn2 has also been suggested to regulate intracellular ROS via regenerating hyperoxidized peroxiredoxins in renal proximal tubule cells (Yang et al., 2014). Contrarily, Sesn2-knockdown in renal proximal tubule cells significantly decreased hyperoxidized peroxiredoxin production and resulted in ROS accumulation (Yang et al., 2014). These studies indicated that Sesn2 could exert evident anti-oxidant effects in various diseases. Our study demonstrated that Sesn2 overexpression could attenuate oxidative stress in pathological cardiac hypertrophy via increasing Nrf2/HO-1 signaling pathway and SOD activity but decreasing the production of MDA, p67, and 4-HEN and depressing NADPH activity. What’s more, this study first found that Sesn2 overexpression failed to exert its antioxidant function in AMPKα2-/- mice.

Theoretically, there are three possibilities to explain this phenomenon. Firstly, Sesn2-dependent AMPK activation and mTORC1 inhibition could be essential for maintaining basal autophagy, which could help organism get rid of dysfunctional mitochondria by preventing electron leak and excessive ROS production (Packer, 2020). Lee et al. demonstrated that dSesn deletion in Drosophila could cause mitochondrial dysfunction and ROS production in skeletal muscle and heart. However, pharmacological mTORC1 inhibitors could completely mitigate excessive ROS production in dSesn deficient muscle. Moreover, the mutant of dSesn in its antioxidant domain remained to suppress ROS accumulation by its ability to depress mTORC1 activity. These studies indicated that the antioxidant property of sestrin might be associated with its regulation on mTORC1. Secondly, some studies have also indicated that activated AMPK could promote Nrf2 expression to enhance antioxidant functions in various animal disease models (Fischhuber et al., 2020; Huang et al., 2020). Moreover, Manuel et al. (Matzinger et al., 2020) demonstrated that AMPKα1 directly phosphorylated Nrf2 at serine 374, 408, and 433 to determine the extent of transactivation of Nrf2-regulated downstream genes (Matzinger et al., 2020). Zhou et al. (Li et al., 2020) exhibited that sulforaphane (SFN) treatment protected against type-2-diabetes-induced renal lipotoxicity through AMPKα2-mediated Nrf2 activation and the beneficial effects of SFN were lost in AMPKα2-/- mice (Li et al., 2020). These studies indicated that AMPKα2 activation could promote Nrf2 mediated antioxidant function. Thirdly, AMPK has also been suggested to regulate NADPH oxidase activity. In high glucose-treated podocytes, AMPK inactivation led to upregulation of Nox4 and enhancement of NADPH oxidase and thus resulted in podocyte apoptosis (Eid et al., 2010). Pharmacologic activation of AMPK significantly depressed Nox4 expression and alleviated oxidative stress (Eid et al., 2010). Assaad AE et al. (Eid et al., 2013) further demonstrated that Sesn2 mediated AMPK activation alleviated HG-induced fibronectin synthesis via blocking Nox4-dependent ROS and peroxynitrite production in glomerular mesangial cells (Eid et al., 2013). Our study also demonstrated that Sesn2 overexpression could suppress Nox4 expression in hypertrophic mouse hearts. Therefore, previous published studies support our finding that Sesn2 overexpression in cardiomyocytes could no longer depress oxidative stress in AMPKα2-/- mice hearts. The regulation of AMPK-mTORC1 signaling pathway by Sesn2 might be more potent than other anti-oxidants in depressing ROS overproduction in pathological cardiac hypertrophy.

## 5 Conclusion

This study first demonstrated that Sesn2 overexpression (at 3, 6, and 9 folds) showed no genetic toxicity in transgenic mice hearts. Sesn2 overexpression could mitigate pressure overload-induced cardiac hypertrophy *in vivo* and Ang II-induced NRCMs hypertrophy *in vitro via* activating AMPKα2, depressing ACC1/mTORC1 signaling, and oxidative stress, as well as restoring Nrf2/HO-1 signaling. We also showed that Sesn2 mediated AMPKα2 activation might be the key point for its activity in preventing excessive ROS accumulation and restoring Nrf2/HO-1 signaling. Strategies on regulating Sesn2 expression by genetic or pharmacologic means might be effective for preventing pathological cardiac hypertrophy and heart failure.

## Data Availability

The original contributions presented in the study are included in the article/Supplementary Materials, further inquiries can be directed to the corresponding author.
