# Supplementary material for: Knockout of AMPKα2 Blocked the Protection of Sestrin2 Overexpression Against Cardiac Hypertrophy Induced by Pressure Overload
Source: Front Pharmacol. 2021 Nov 17;12:716884. doi: 10.3389/fphar.2021.716884 (PMC8635785; doi:10.3389/fphar.2021.716884)

Figure 1

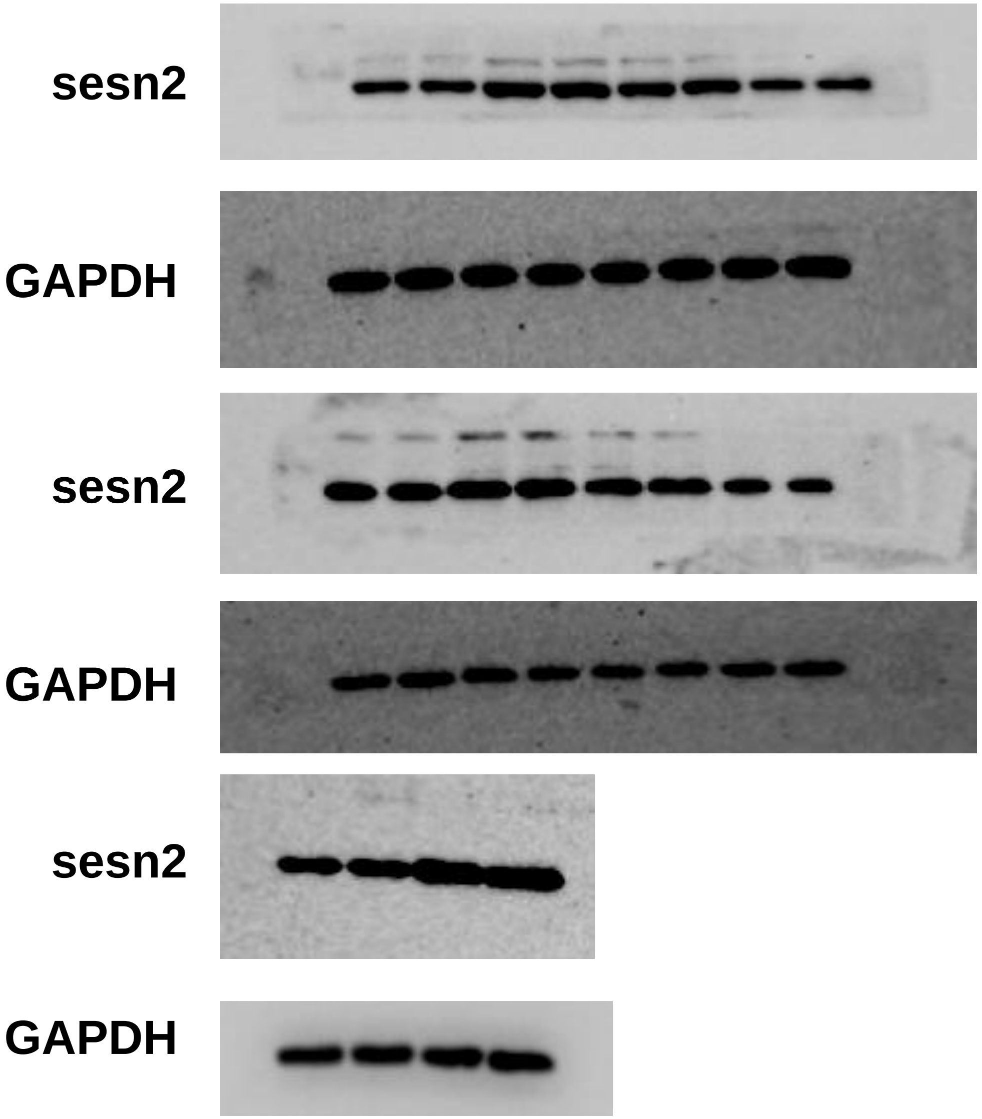

Figure 2

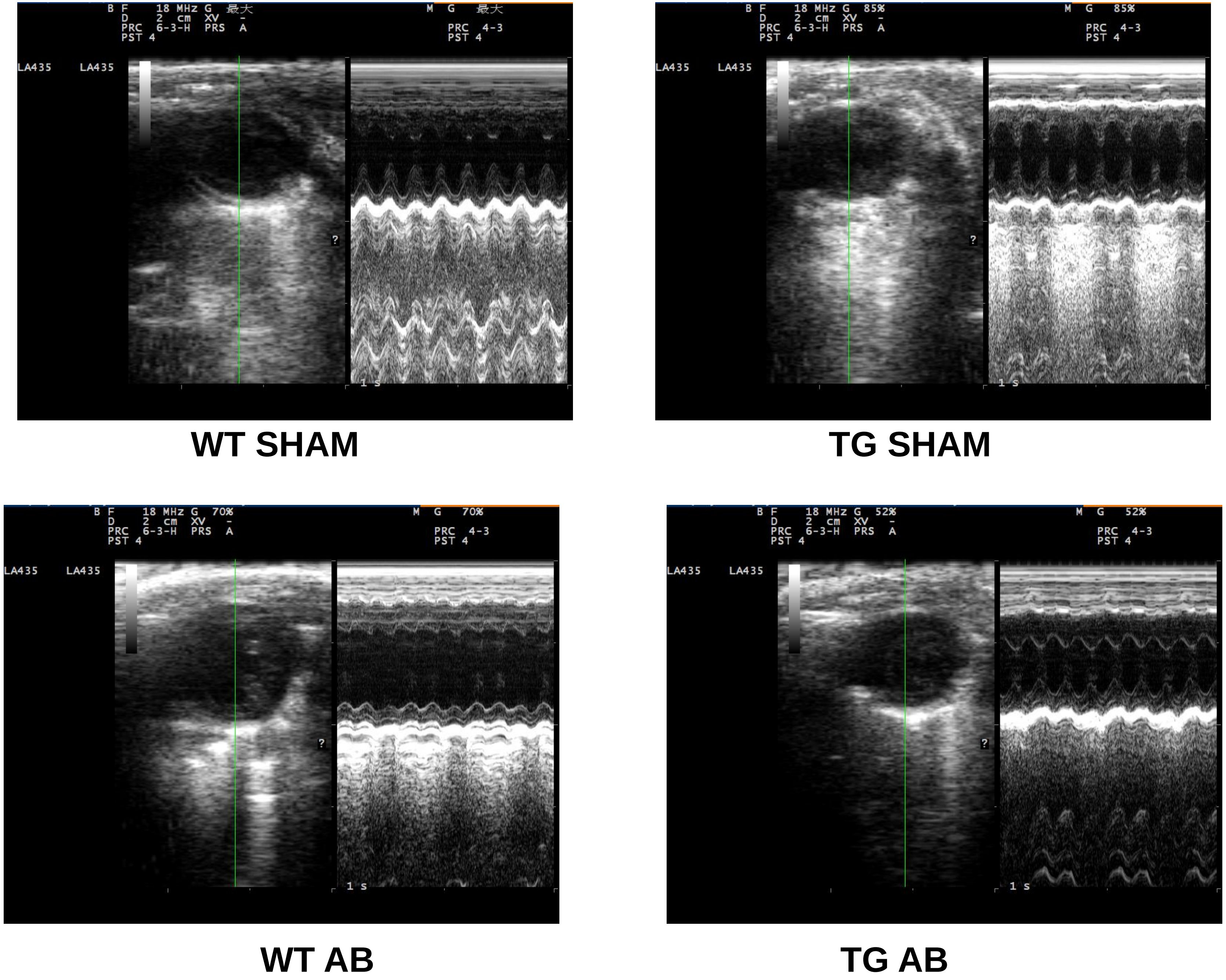

Figure 3

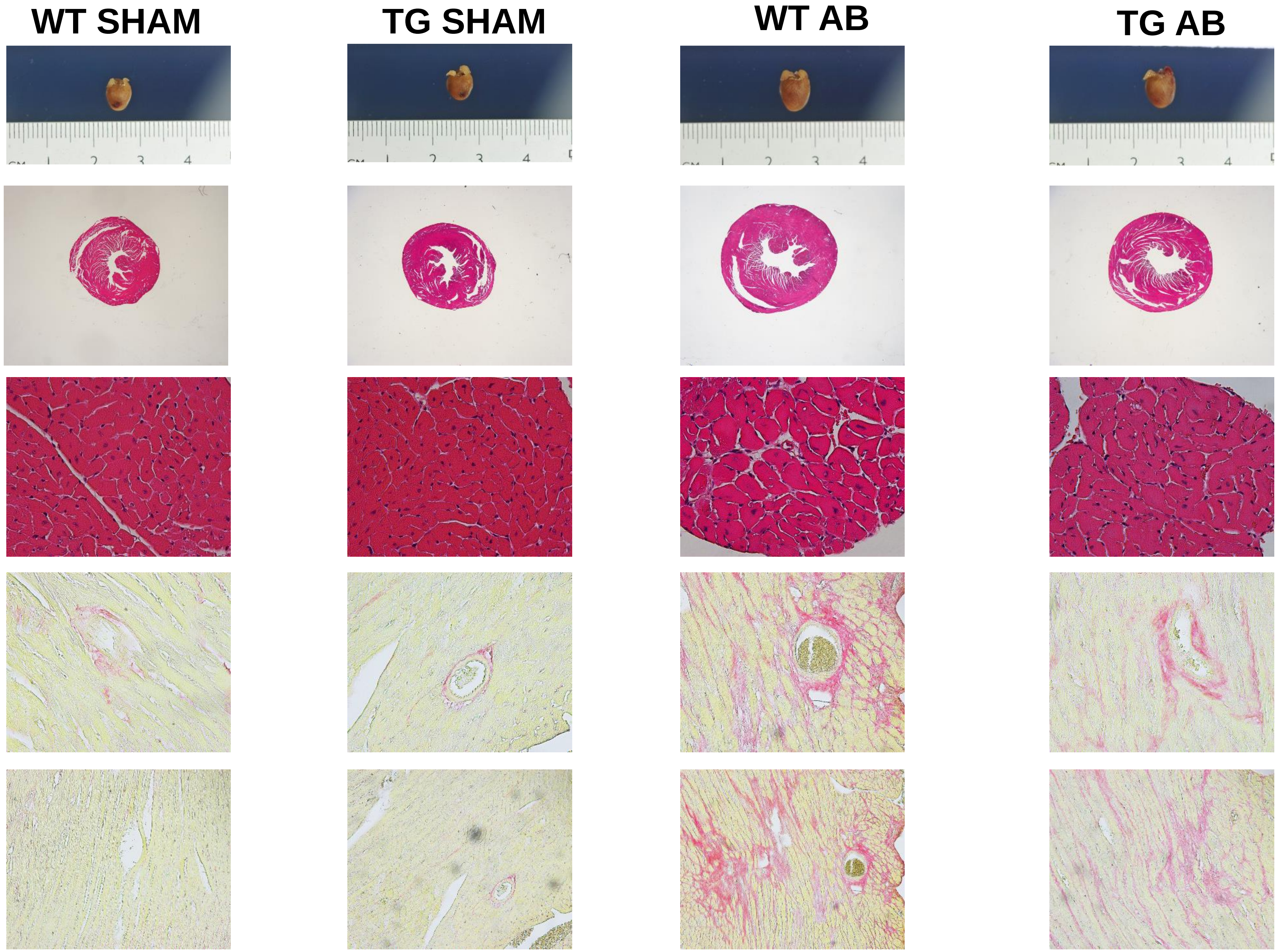

Figure 4

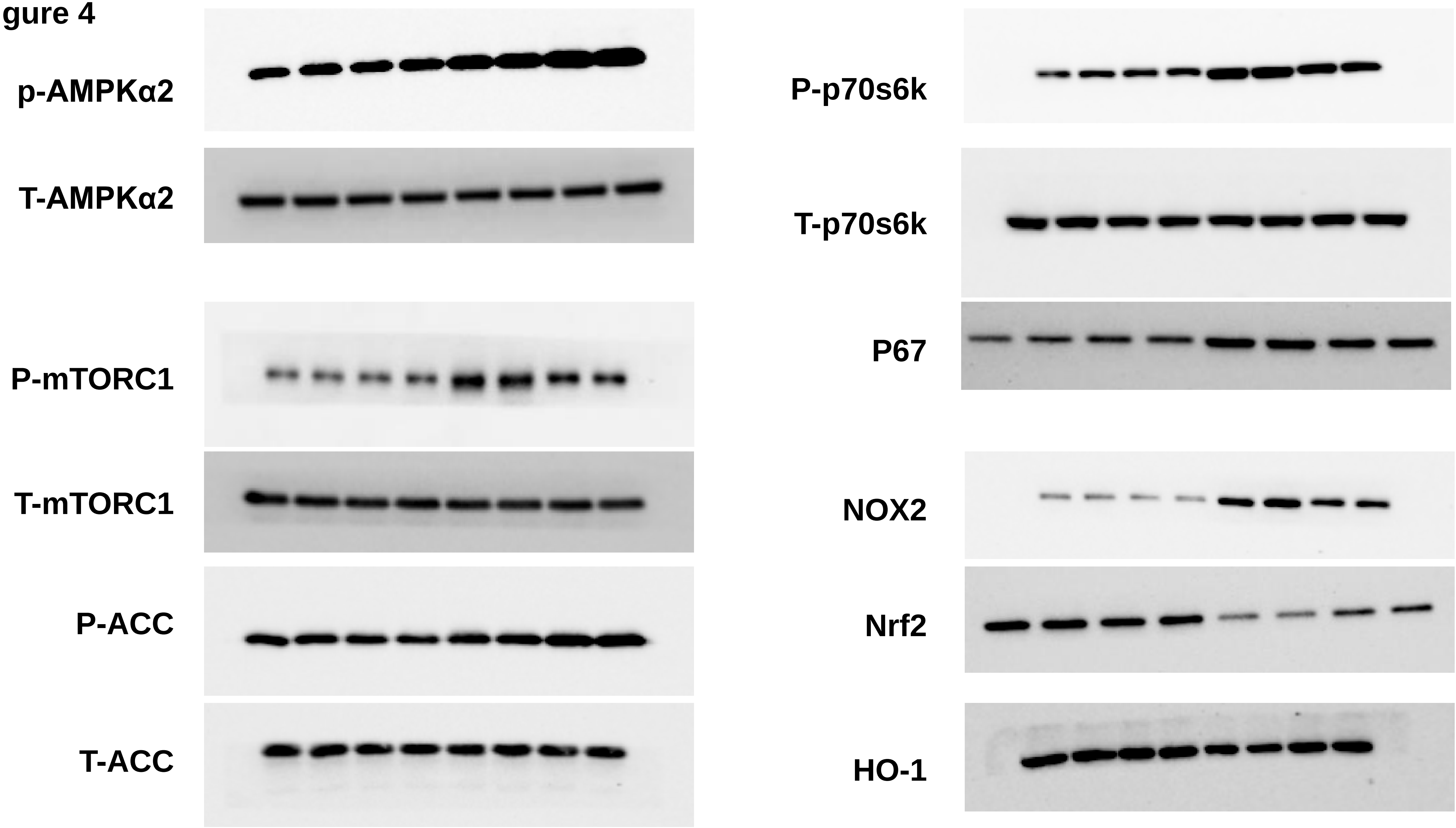
p-AMPKα2
T-AMPKα2
P-mTORC1
T-mTORC1
P-ACC
T-ACC
P-p70s6k
T-p70s6k
P67
NOX2
Nrf2
HO-1

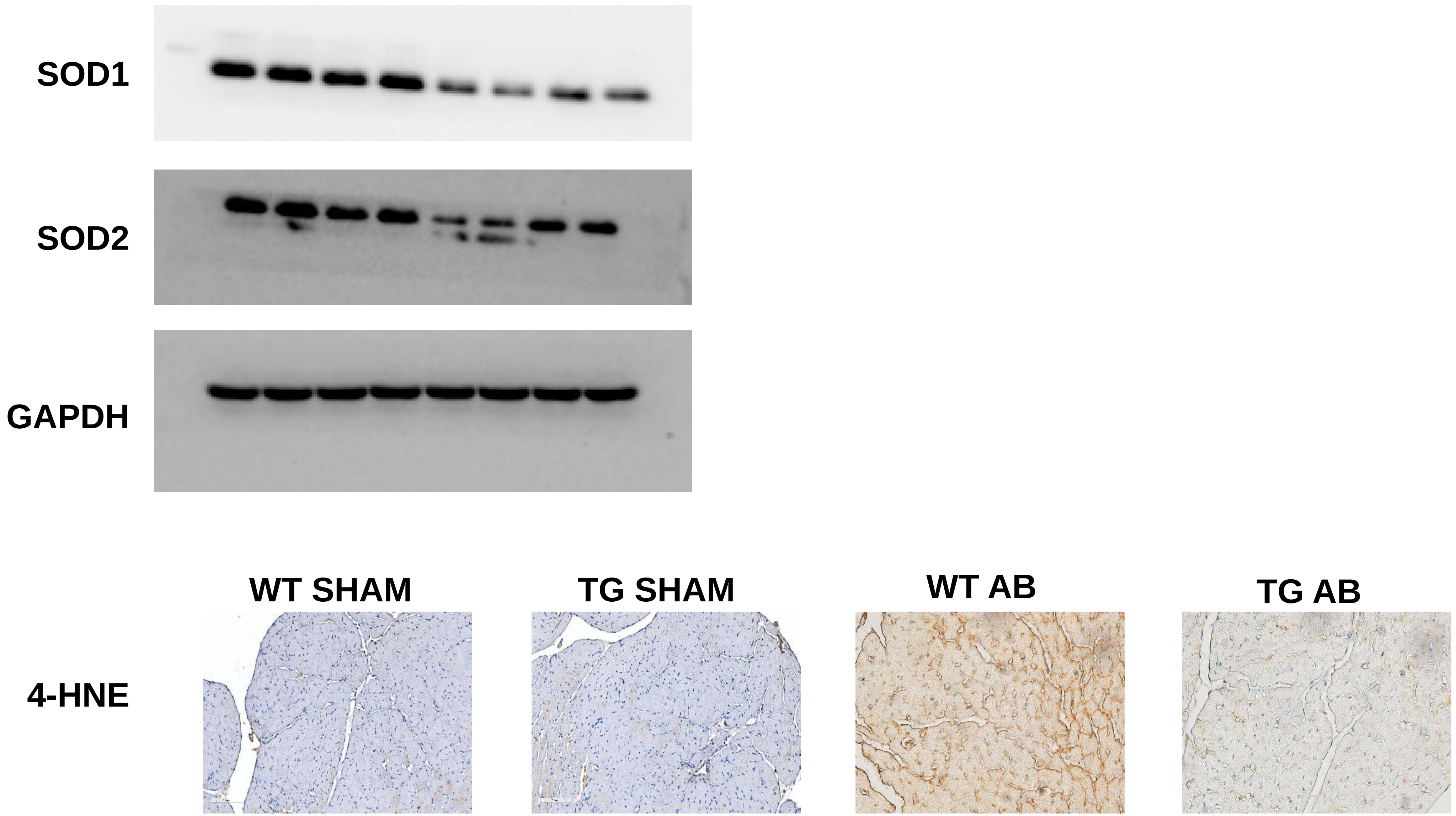
SOD1
SOD2
GAPDH
WT SHAM
TG SHAM
WT AB
TG AB
4-HNE

Figure 5

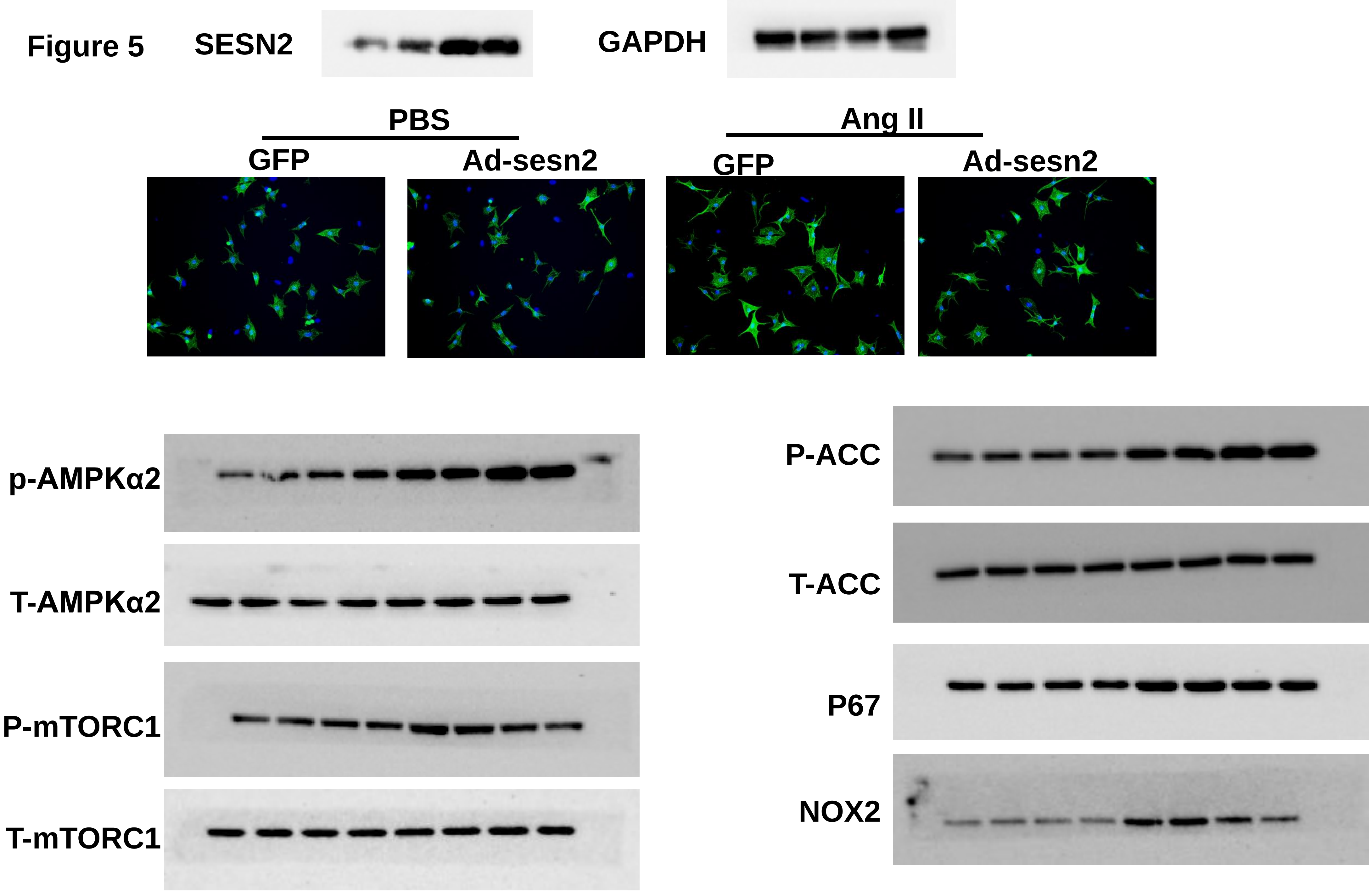

SESN2
GAPDH
PBS
Ang II
GFP
Ad-sesn2
GFP
Ad-sesn2
p-AMPKα2
T-AMPKα2
P-mTORC1
T-mTORC1
P-ACC
T-ACC
P67
NOX2

Figure 5

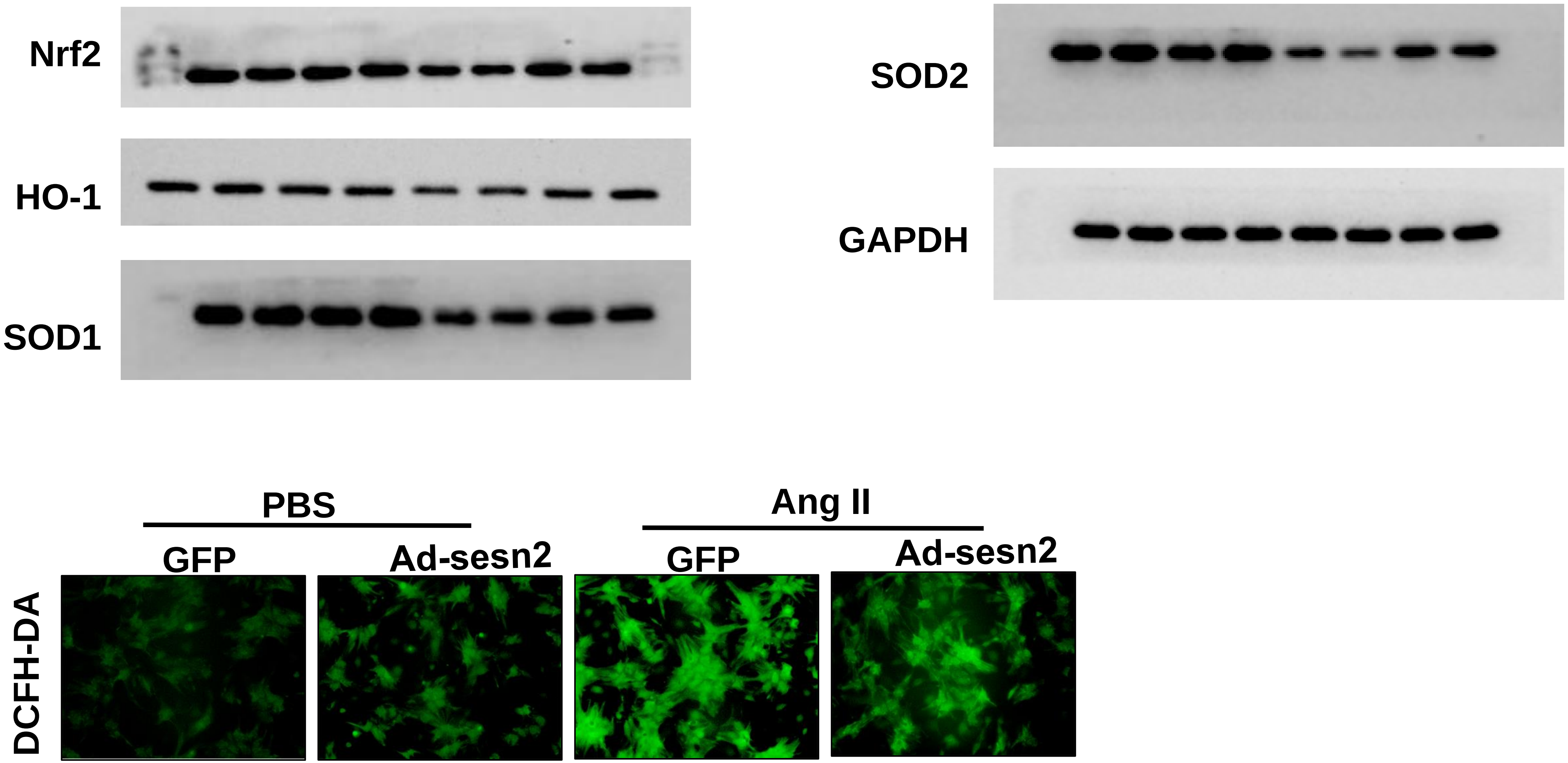
Nrf2
HO-1
SOD1
SOD2
GAPDH
PBS
Ang II
GFP
Ad-sesn2
GFP
Ad-sesn2
DCFH-DA

Figure 6

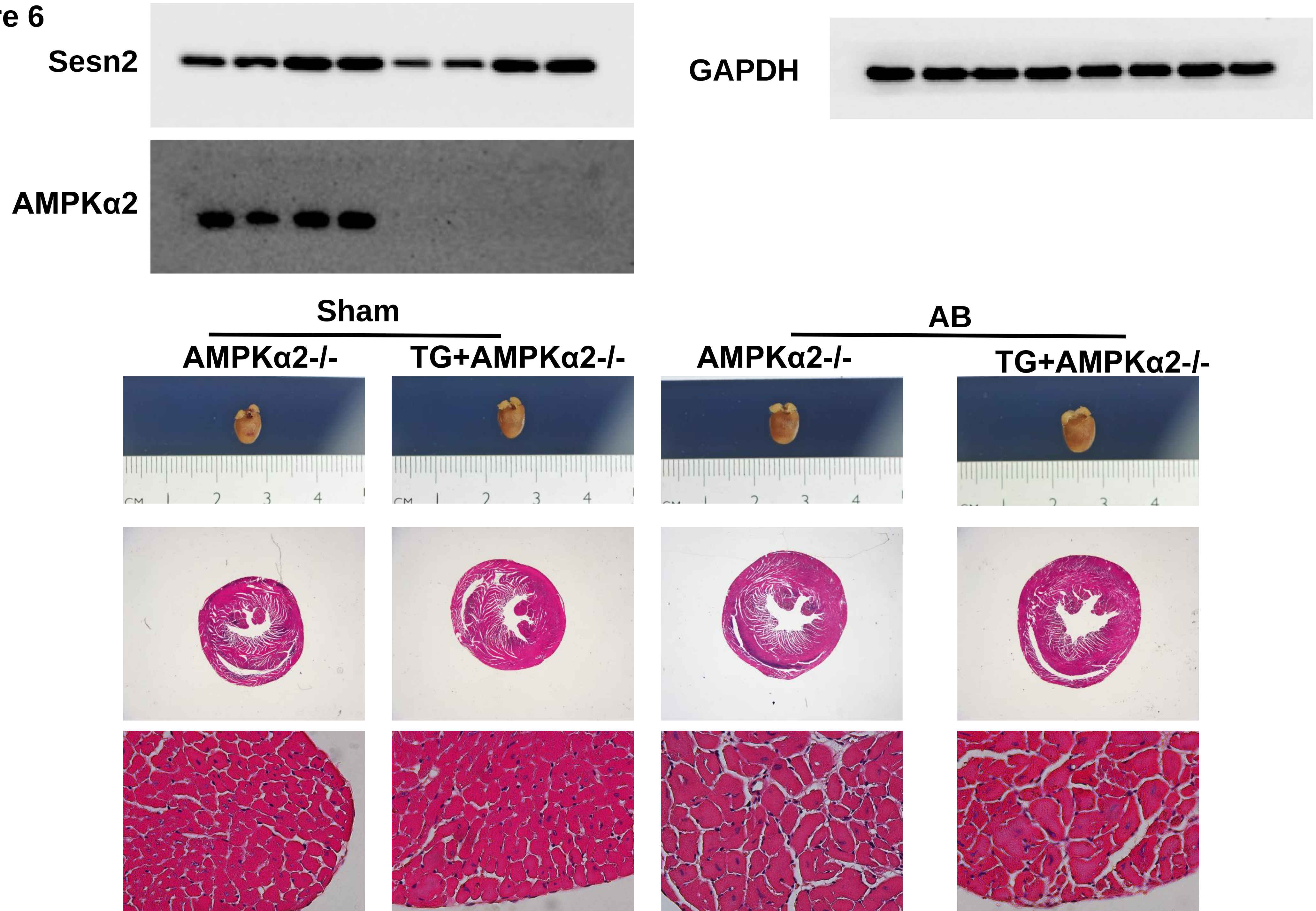
Sesn2
GAPDH
AMPKα2
Sham
AB
AMPKα2-/-
TG+AMPKα2-/-
AMPKα2-/-
TG+AMPKα2-/-

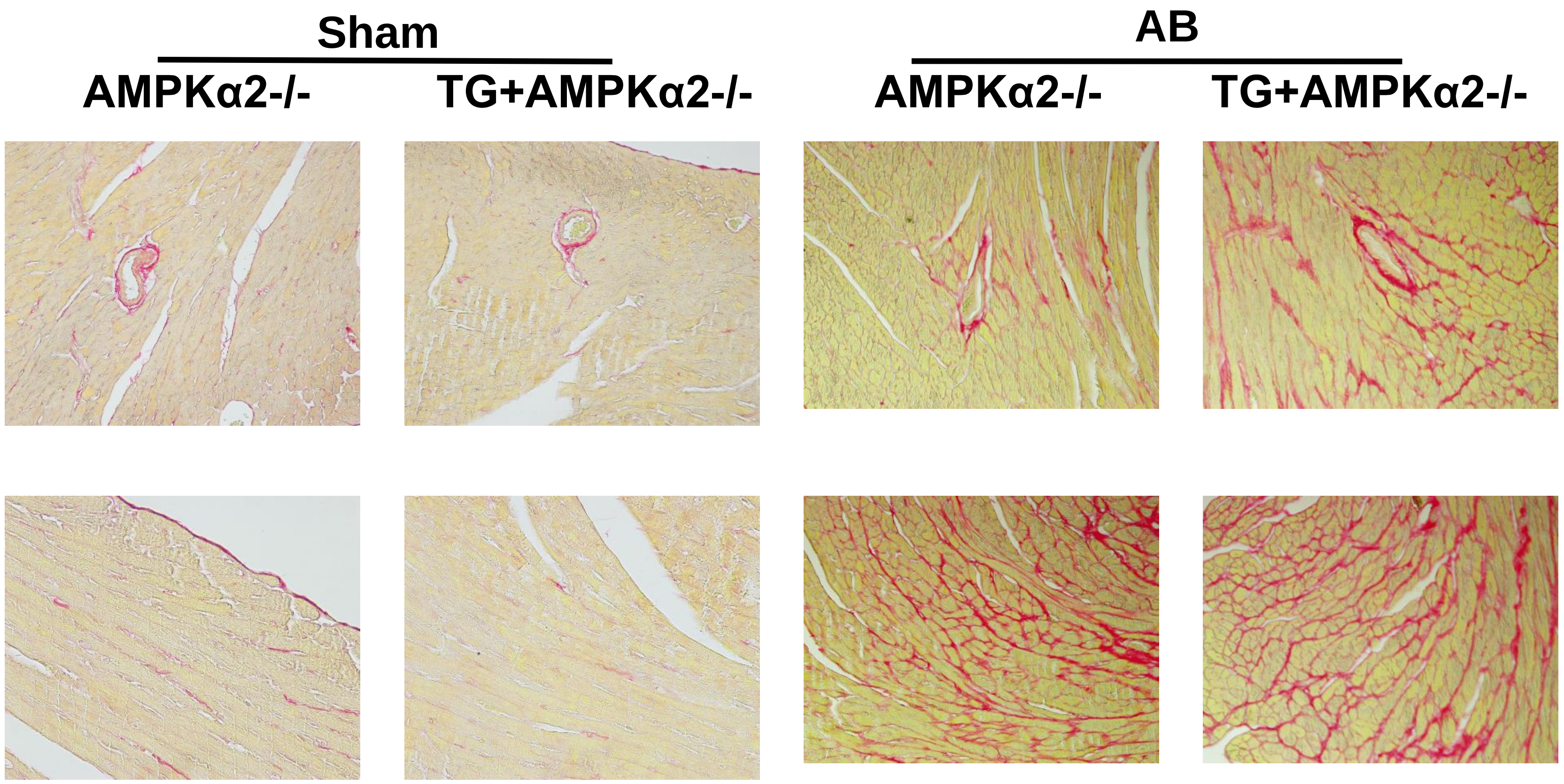
Sham
AB
AMPKα2-/-
TG+AMPKα2-/-
AMPKα2-/-
TG+AMPKα2-/-

S figure 2

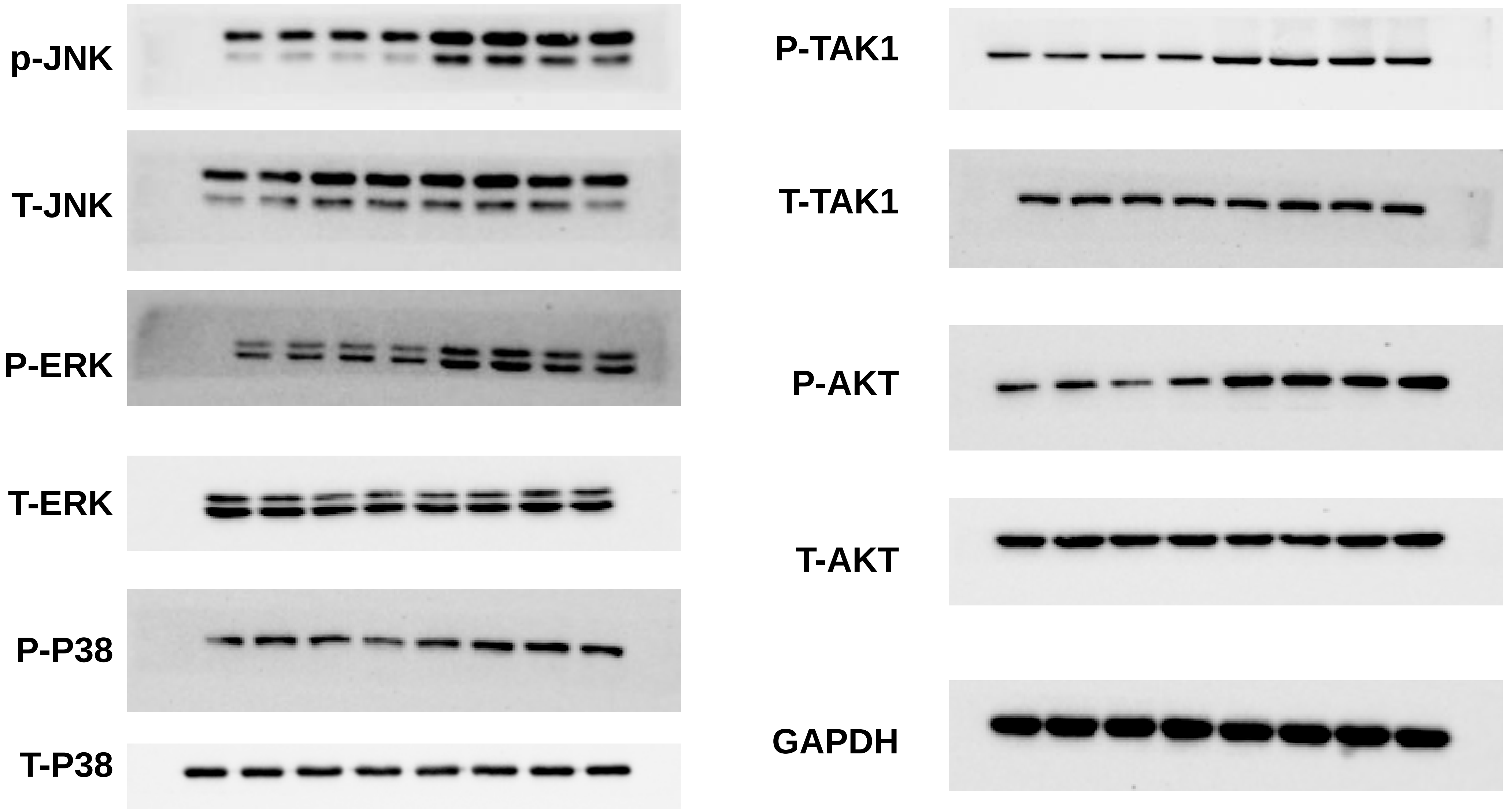

S figure 3

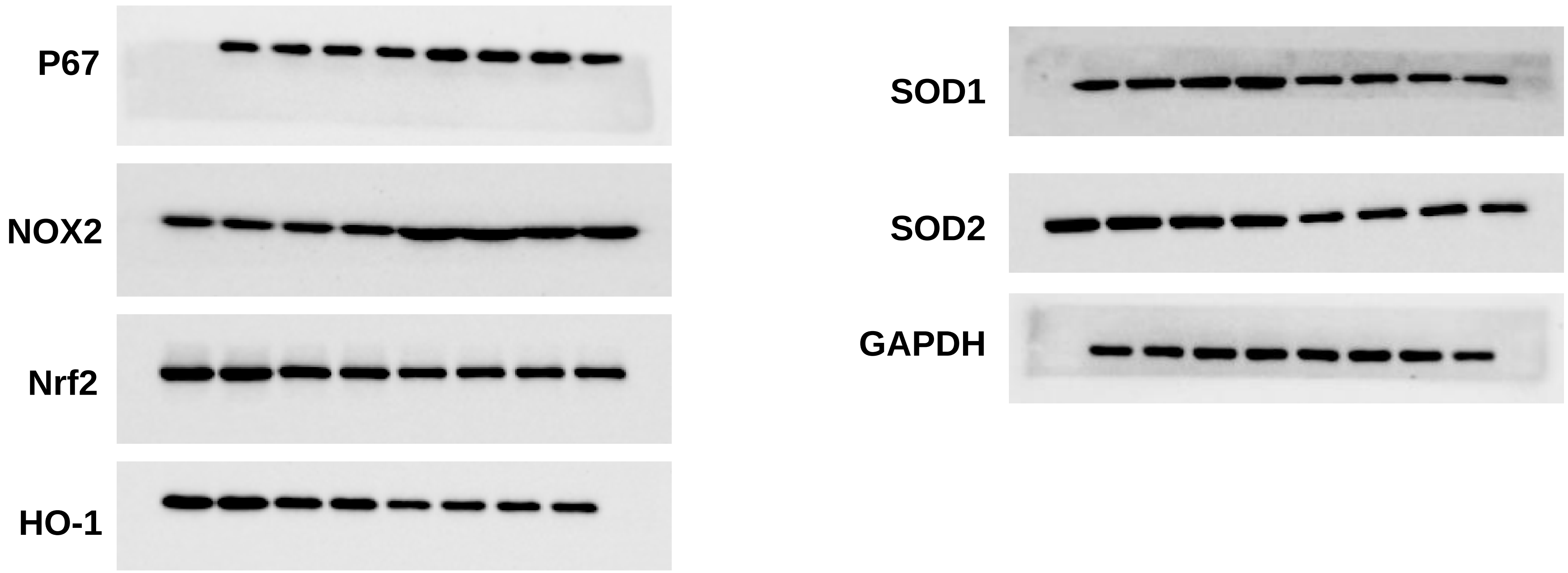

S figure 4

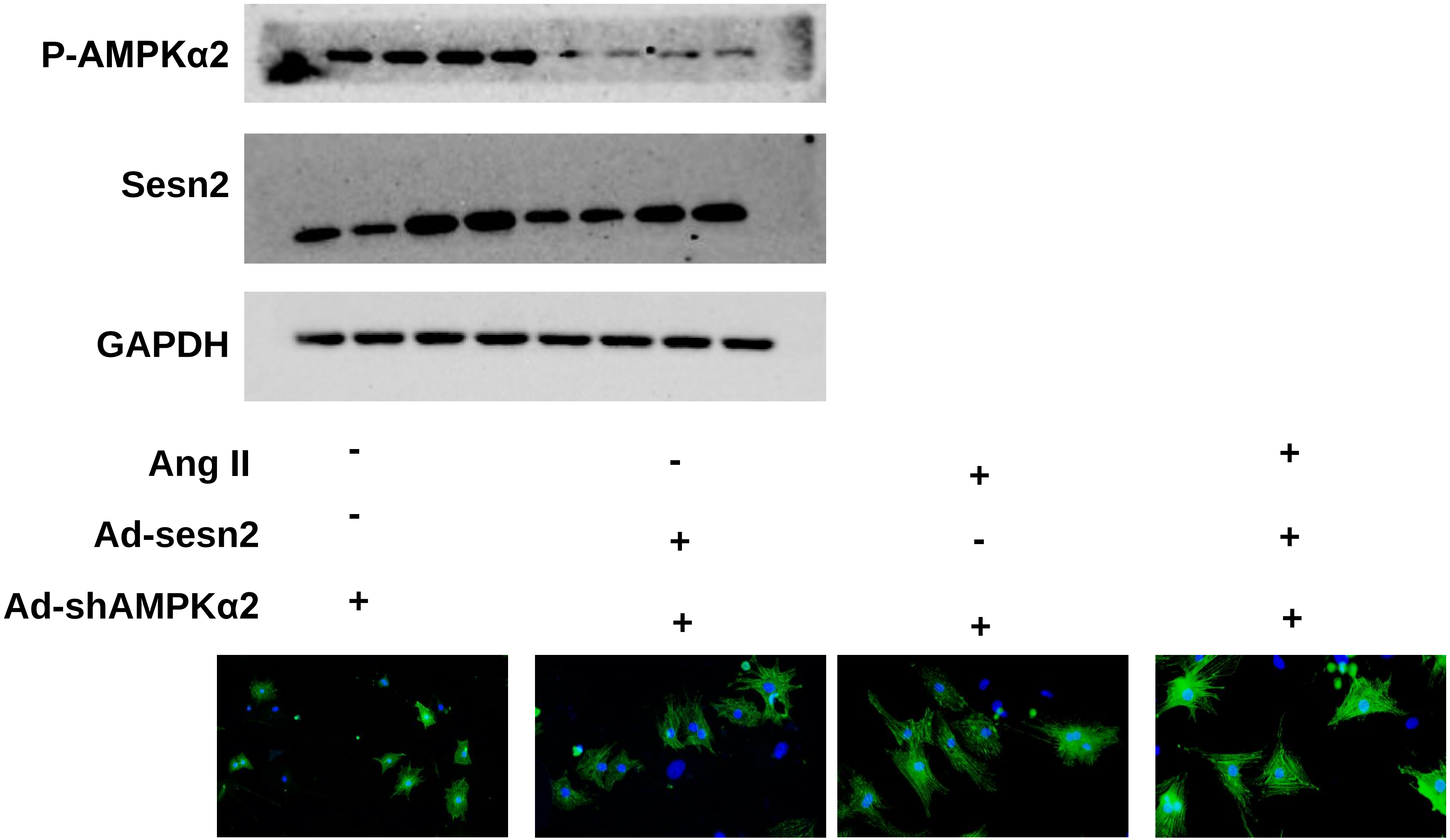

Supplement: Supplementary file 1 [file DataSheet2.PDF]
