# Supplementary material for: Knockout of AMPKα2 Blocked the Protection of Sestrin2 Overexpression Against Cardiac Hypertrophy Induced by Pressure Overload
Source: Front Pharmacol. 2021 Nov 17;12:716884. doi: 10.3389/fphar.2021.716884 (PMC8635785; doi:10.3389/fphar.2021.716884)
Supplement: Supplementary file 2 [file DataSheet1.PDF]

Supplementary Material

Supplementary Figures

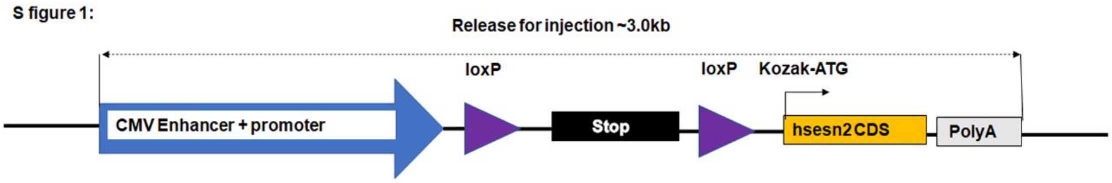

Supplementary Figure 1. Schematic diagram depicting the construction of Sesn2-CTG experimental mice.

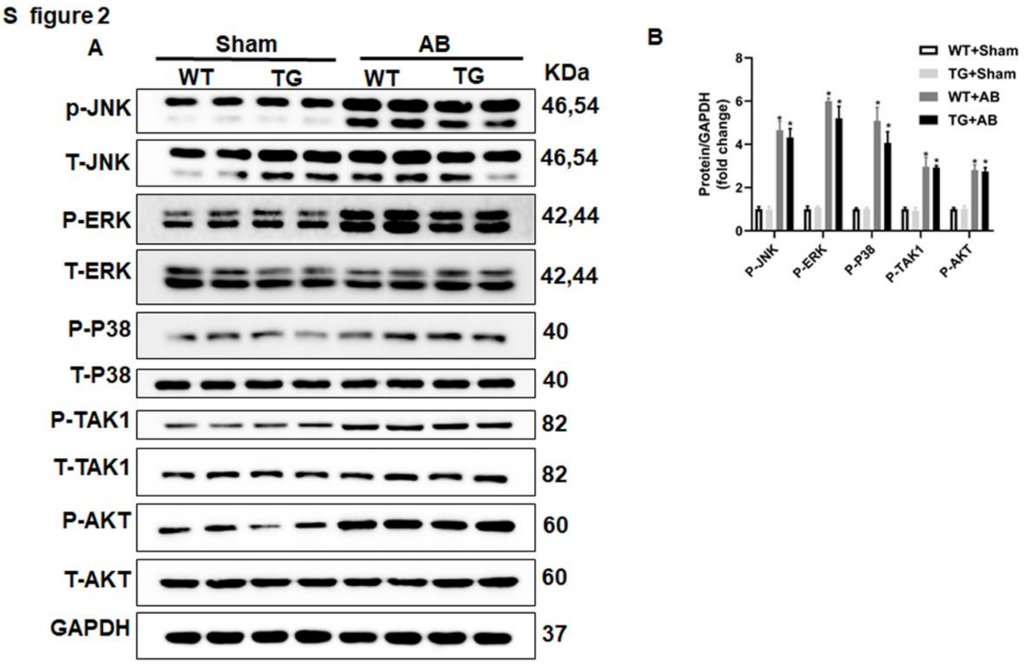

Supplementary Figure 2. Sesn2 overexpression did not affected MAPK, TAK1 and AKT pathway. A. Representative western blots of the phosphorylated and total protein expression levels of JNK, ERK, P38, TAK1 and AKT in cardiac tissues of sesn2-TG and WT mice 8 weeks after sham or AB surgery (n=6). B. Quantification of the proteins. Data were described as mean ± SD. *$P < 0.05$

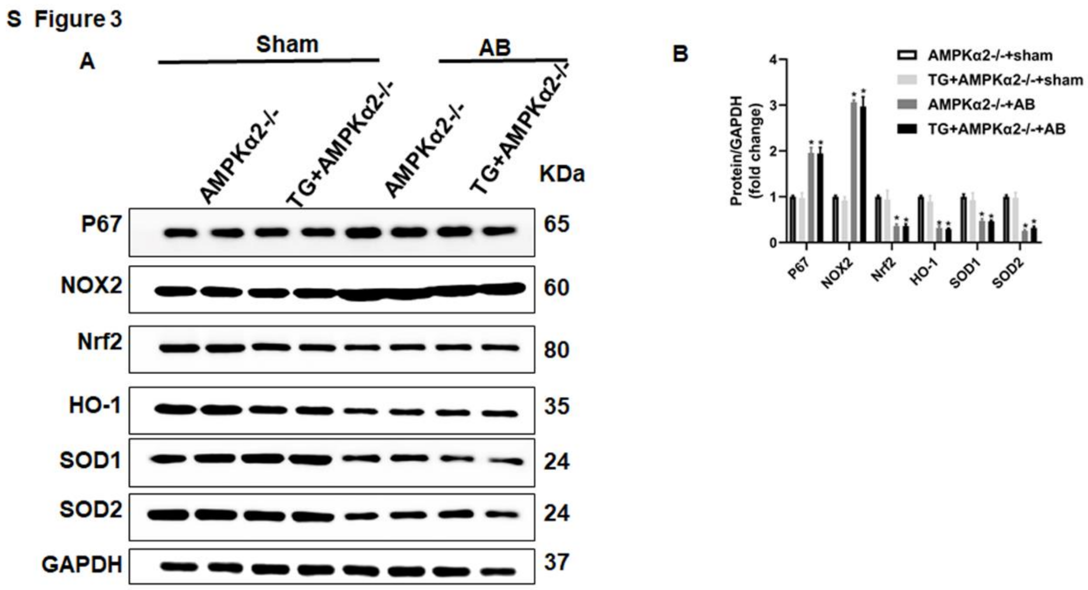

Supplementary Figure 3. Sesn2 overexpression could not suppressed oxidative stress in AMPKα2 knockout mice. A-B. Representative western blots and quantitative results of P67, NOX2, Nrf2, HO-

1, SOD1 and SOD2 in cardiac tissues of mice 8 weeks after sham or AB surgery in different groups. (n = 6). Data were described as mean ±SD. *P < 0.05

S Figure 4

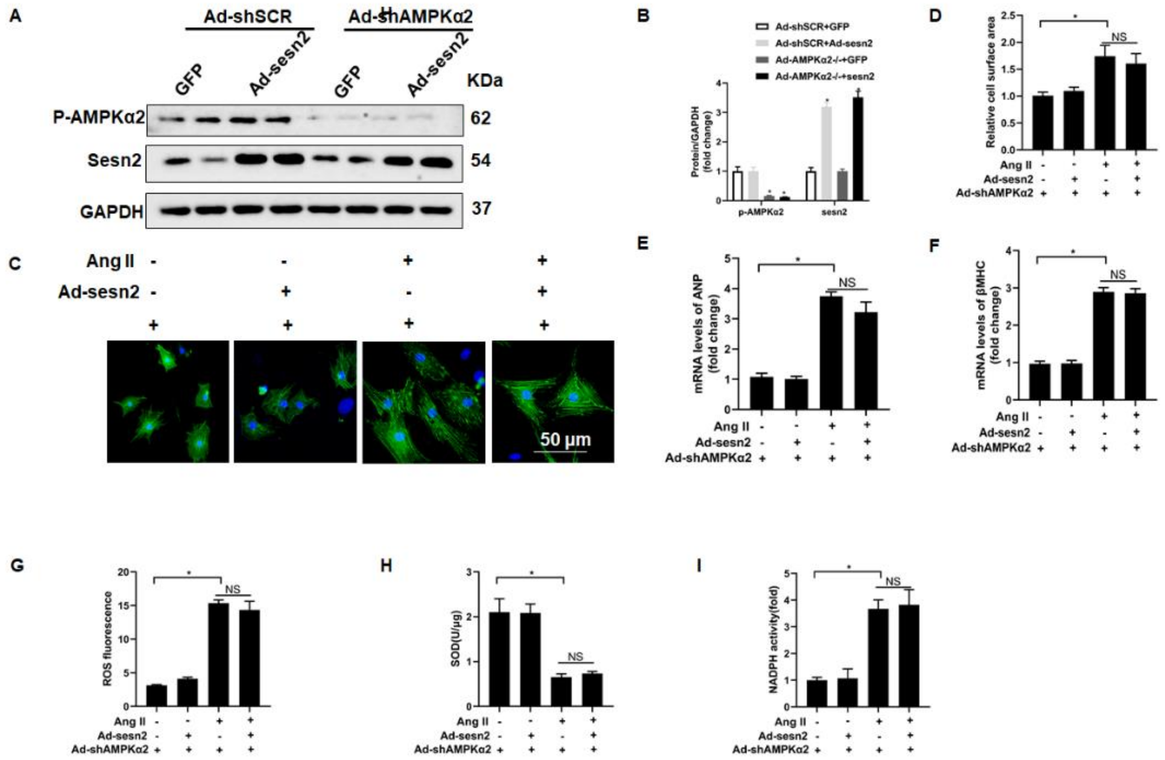

Supplementary Figure 4. Sesn2 overexpression could not prevent Ang II induced hypertrophy and oxidative stress in AMPKα2-silent NRCMs. NRCMs were transfected with Ad-sesn2 and Ad-shAMPKα and then stimulated with Ang II for 48h. A-B. Representative western blots and quantitative results of P-AMPKα2 and sesn2 in NRCMs transfected with Ad-shRNA or Ad-shAMPKα. (n=6 independent experiments). C-D. Representative images of α-actinin staining and statistical results of cell surface areas in the indicated groups. (n ⩾ 100 cells per group). E-F. Expression of mRNA of hypertrophic markers in each group (n = 6 independent experiments). E. ANP; F. βMHC; G-I. ROS levels and SOD and NADPH oxidase activities in the indicated groups (n = 6). Data were described as mean ±SD. *P < 0.05.
